# K63-polyubiquitinated HAUSP deubiquitinates HIF-1α and dictates H3K56 acetylation promoting hypoxia-induced tumour progression

**DOI:** 10.1038/ncomms13644

**Published:** 2016-12-09

**Authors:** Han-Tsang Wu, Yi-Chih Kuo, Jung-Jyh Hung, Chi-Hung Huang, Wei-Yi Chen, Teh-Ying Chou, Yeh Chen, Yi-Ju Chen, Yu-Ju Chen, Wei-Chung Cheng, Shu-Chun Teng, Kou-Juey Wu

**Affiliations:** 1 Research Center for Tumor Medical Science, Graduate Institutes of Biomedical Sciences and New Drug Development, China Medical University, Taichung 404, Taiwan; 2 Institute of Clinical Medicine, National Yang-Ming University, Taipei 112, Taiwan; 3 Division of Thoracic Surgery, Department of Surgery, Taipei Veterans General Hospital, Taipei 112, Taiwan; 4 Taiwan Advance Biopharm (TABP), Inc., Xizhi city, New Taipei City 221, Taiwan; 5 Institute of Biochemistry & Molecular Biology, National Yang-Ming University, Taipei 112, Taiwan; 6 Department of Pathology, Taipei Veterans General Hospital, Taipei 112, Taiwan; 7 Department of Biotechnology, Hungkuang University, Taichung 433, Taiwan; 8 Institute of Chemistry, Academia Sinica, Taipei 115, Taiwan; 9 Graduate Institute of Microbiology, College of Medicine, National Taiwan University, Taipei 100, Taiwan; 10 Department of Medical Research, China Medical University Hospital, Taichung 404, Taiwan

## Abstract

Intratumoural hypoxia induces HIF-1α and promotes tumour progression, metastasis and treatment resistance. HIF-1α stability is regulated by VHL-E3 ligase-mediated ubiquitin-dependent degradation; however, the hypoxia-regulated deubiquitinase that stabilizes HIF-1α has not been identified. Here we report that HAUSP (USP7) deubiquitinase deubiquitinates HIF-1α to increase its stability, induce epithelial-mesenchymal transition and promote metastasis. Hypoxia induces K63-linked polyubiquitinated HAUSP at lysine 443 to enhance its functions. Knockdown of HAUSP decreases acetylation of histone 3 lysine 56 (H3K56Ac). K63-polyubiquitinated HAUSP interacts with a ubiquitin receptor CBP to specifically mediate H3K56 acetylation. ChIP-seq analysis of HAUSP and HIF-1α binding reveals two motifs responsive to hypoxia. HectH9 is the E3 ligase for HAUSP and a prognostic marker together with HIF-1α. This report demonstrates that hypoxia-induced K63-polyubiquitinated HAUSP deubiquitinates HIF-1α and causes CBP-mediated H3K56 acetylation on HIF-1α target gene promoters to promote EMT/metastasis, further defining HAUSP as a therapeutic target in hypoxia-induced tumour progression.

## Abstract (web summary)

Hypoxia-induced transcriptional responses mediated by HIF-1a are regulated through the ubiquitin-dependent pathway to control HIF-1a stability. Here the authors show that the deubiquitinase HAUSP modulates the stability of HIF-1a and K63-polyubiquitinated HAUSP serves as an anchor for HIF-1a-induced gene transcription.

Mammalian cells constantly encounter hypoxic stress and stabilization of hypoxia-inducible factor-1α (HIF-1α) is one of the mechanisms developed by cells to cope with this stress^1^,^2^. Intratumoural hypoxia promotes tumour progression, metastasis and treatment resistance^3^,^4^. Hypoxia/HIF-1α promotes epithelial-mesenchymal transition (EMT), a process that plays a critical role in promoting metastasis by enhancing cancer cell motility and increasing chemoresistance^5^,^6^. The EMT induced by hypoxia is usually regulated by EMT transcriptional regulators, including Snail, Twist1, ZEB1, and so on^3^. HIF-1α stability is mainly regulated at the post-translational levels through ubiquitination^7^,^8^. Under normoxia, HIF-1α is prolyl hydroxylated at amino acid 402 and 564 positions^9^ and the hydroxylated HIF-1α is recognized by von Hippel Lindau (VHL) protein, a component of the ubiquitin E3 ligase complex containing Cul-2, VHL, elongin B and elongin C^10^,^11^. HIF-1α is subsequently ubiquitinated and degraded by the proteasome^7^,^8^. However, HIF-1α proteins can accumulate under normoxia through different mechanisms other than post-translational modification^12^,^13^,^14^.

HAUSP (USP7) is a USP type deubiquitinase that was originally shown to stabilize p53 (ref. 15). However, HAUSP also stabilizes MDM2 and manifests an oncogenic function^16^,^17^. HAUSP deubiquitinates PTEN to cause its nuclear exclusion, leading to tumour aggressiveness^18^. Specific anti-HAUSP inhibitors that can provide therapeutic value for different human cancers were developed^19^,^20^,^21^,^22^. These results indicate that HAUSP may play a crucial role in tumour progression. Whether HAUSP displays a function other than deubiquitinase to mediate tumour progression is unknown.

Lysine-63 (K63)-linked polyubiquitination is shown to have non-proteolytic functions including protein trafficking, kinase and phosphatase activation, DNA repair, NF-kB activation, chromatin dynamics, and so on refs 23, 24, 25, 26. K63-linked polyubiquitinated signalling molecules that play a significant role in signal transduction occur in NF-kB, T-cell receptor, Toll-like receptor, RIG-1-like receptor, NOD-like receptor, DNA damage response pathways and Akt activation^24^,^25^. Among these examples, K63-linked polyubiquitin chains serve as a scaffold to facilitate assembly of a protein complex^27^. Novel functions of the K63 polyubiquitin chains remain to be demonstrated.

Eukaryotic gene transcription starts from the formation of a preinitiation complex that is anchored by the Mediator complex^28^,^29^. The Mediator complex serves as an integrative hub to accommodate transcription factors, co-regulators and the elongation complex to carry out various aspects of transcription including initiation, elongation and RNA processing, which also modulates chromatin architecture^28^,^29^. For HIF-1α-induced gene transcription, the CDK8-Mediator complex module facilitates the transcriptional elongation of HIF-1α target genes^30^. However, whether there is a master scaffold that can receive the inputs from HIF-1α, CBP (a co-activator of HIF-1α), the CDK8-Mediator complex, and the elongation complex under hypoxia and convert them into fully active HIF-1α-induced gene transcription remains to be demonstrated.

Acetylated histone H3K56 (H3K56Ac) has been shown to play a critical role in the packaging of DNA into chromatin following DNA replication and repair in budding yeast, Drosophila and humans^31^,^32^,^33^. H3K56Ac is also involved in cell proliferation, cancer and embryonic stem cell pluripotency transcription^34^,^35^. H3K56 is acetylated by Rtt109 from budding yeast and by CBP/p300 in Drosophila and humans^32^,^34^. However, CBP/p300 can also mediate acetylation of H3K18 and H3K27 in nuclear receptor transactivation^36^. The event to determine specific acetylation of H3K56 or H3K18/27 (that is, CBP-substrate selectivity) is largely unknown.

In this report, HAUSP is identified as an HIF-1α deubiquitinase. Hypoxia-induced post-translational modification of HAUSP is crucial for HAUSP to carry out its functions and dictates a specific histone modification to regulate HIF-1α target gene transcription. These results provide a unique mechanism for hypoxia-induced tumour progression through post-translational and epigenetic regulations.

## Results

### HAUSP interacts with and deubiquitinates HIF-1α

**Figure 1:**
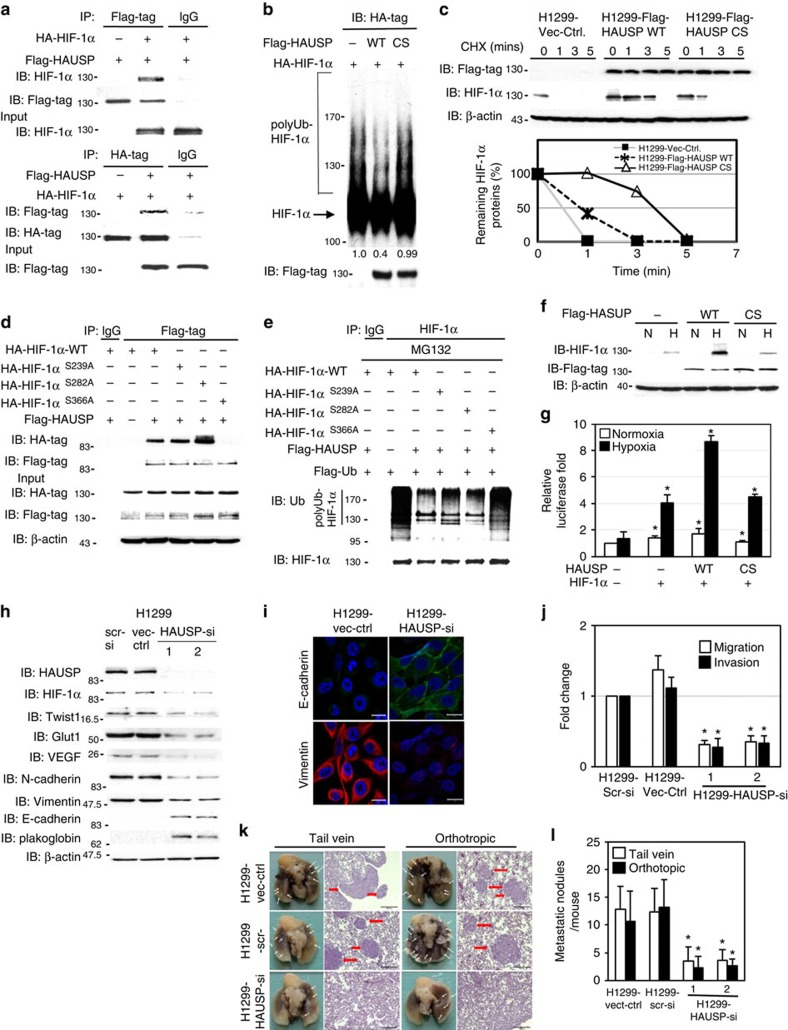
HAUSP stabilizes HIF-1α to modulate its activity and knockdown of HAUSP reverses EMT and decreases metastatic activity. (**a**) Interaction between HAUSP and HIF-1α using co-immunoprecipitation assays. (**b**) *In vitro* deubiquitination assays showed that HAUSP significantly decreased the levels of polyubiquitinated HIF-1α. The densitometer reading of polyubiquitinated HIF-1α levels was listed underneath each lane. The first lane was arbitrarily set as 1. (**c**) Stably expressed HAUSP in H1299 cells significantly increased the half-life of HIF-1α. Lower panel: the degradation curve by densitometric measurement to determine the half-life of endogenous HIF-1α proteins. (HAUSP-CS mutant: negative control). (**d**) The HIF-1α^S366A^ mutant did not interact with HAUSP in co-immunoprecipitation experiments in 293T cells. (**e**) The polyubiquitinated HIF-1α^S366A^ mutant could not be deubiquitinated by HAUSP. (**f**) Western blot analysis showed the increased HIF-1α levels through co-expression of wild-type HAUSP in 293T cells. β-actin, loading control; N, normoxia; H, hypoxia. (**g**) Further activation of the *Twist1* promoter-driven reporter activity by co-expressing HIF-1α and HAUSP in transient transfection assays. The samples of transfecting with reporter alone (normoxia or hypoxia) were used as the controls. (**h**) Knockdown of HAUSP reversed EMT marker gene expressions and decreased the expression of HIF-1α downstream targets in H1299 cells that constitutively expressed HIF-1α. (**i**) Immunofluorescence staining showed the reversion of EMT marker gene expression in H1299 cells with HAUSP knockdown. Green fluorescence: E-cadherin, red fluorescence: vimentin. (**j**) Knockdown of HAUSP decreased the *in vitro* migration and invasion activity of H1299 cells. The H1299 scrambled-siRNA control clone was used as the control. (**k**) Representative photos of gross anatomy and histology pictures of H1299 cells (vector control, scrambled siRNA control versus knockdown of HAUSP) injected (tail vein or orthotopic) into nude mice. White arrows indicated tumour nodules. (**l**) The number of metastatic lung tumour nodules of H1299 cells (vector control, scrambled siRNA control versus knockdown of HAUSP). The number of mice used for each group was 6. The scrambled siRNA control was used as the control lane. For (**g**,**j**), data from three independent experiments are expressed as mean±s.d. For (**l**), data are presented as the mean±s.d. (*n*=6 mice/group).The asterisk (*) indicated statistical significance (*P*<0.05) between experimental and control clones or transfections, *t*-test.

Since both HAUSP and HIF-1α play a significant role in tumour progression and aggressiveness^3^,^15^, we evaluated whether HAUSP could deubiquitinate HIF-1α through interacting with HIF-1α. Co-immunoprecipitation experiments showed the interaction between HAUSP and HIF-1α by expressing both proteins in 293T cells (Fig. 1a). Endogenous interaction was exemplified using extracts from H1299 cells under normoxia or hypoxia immunoprecipitated by anti-HAUSP antibodies (Supplementary Fig. 1a). A domain mapping experiment showed that the N-terminal (a.a. 1–400) domain of HIF-1α immunoprecipitated with HAUSP (Supplementary Fig. 1b). The GST pull-down assays showed that the N-terminal domain (a.a. 1–209) of HAUSP directly interacted with the N-terminal (a.a. 1–400) domain of HIF-1α (Supplementary Fig. 1c). This interaction was verified *in vivo* by overexpressing HIF-1α with wild-type or truncated HAUSP (a.a. 210–1100); no interaction was detected between HIF-1α and truncated HAUSP under hypoxia (Supplementary Fig. 1d). These results indicate a direct interaction between specific domains of HAUSP and HIF-1α.

To determine the crucial role of the deubiquitinase activity of HAUSP in HIF-1α deubiquitination, an HAUSP expression vector was transiently overexpressed in 293T cells together with a HIF-1α and an HA-ubiquitin plasmid. Wild-type HAUSP, but not the enzymatically inactive mutant (HAUSP-CS)^15^, decreased the ubiquitinated HIF-1α *in vivo* under MG132 treatment (Supplementary Fig. 1e). De-ubiquitination by wild-type HAUSP, but not by HAUSP-CS, was validated by the *in vitro* deubiquitinase assay (Fig. 1b). The half-life of HIF-1α was significantly prolonged in the lung cancer cell line H1299 stably expressing wild-type HAUSP, but not in cells expressing HAUSP-CS, in the cycloheximide assay, which inhibits protein synthesis (Fig. 1c). Knockdown of HAUSP in H1299 cells increased the polyubiquitinated HIF-1α levels (Supplementary Fig. 1f). Further experiments showed that the a.a. 300–400 domain in HIF-1α was the major domain interacting with HAUSP (Supplementary Fig. 1g,h). Mutational analysis identified the HAUSP recognition consensus sequence (P/AXXS, a.a 363-PVES-366) in HIF-1α^37^. Mutation of the sequence abolished the interaction between HAUSP and HIF-1α and the ability of HAUSP to deubiquitinate HIF-1α (Fig. 1d,e). Co-immunoprecipitation assays were also performed to determine the interactions between HIF-1α and several other members of the DUB family, including USP12, USP14 and USP21. Only HAUSP interacted with HIF-1α (Supplementary Fig. 1i). Further experiments showed that USP21 did not interact with HIF-1α or increase the protein levels of HIF-1α under hypoxia (Supplementary Fig. 1j,k). Finally, co-immunoprecipitation experiments using anti-HIF-1α antibodies to pull down VHL and components of the E3 ubiquitin ligase complex showed that HAUSP knockdown did not affect the interaction between HIF-1α and VHL or the components of the E3 ubiquitin complex (Supplementary Fig. 1l), indicating that the ability of HAUSP to increase HIF-1α stability was independent of its interaction with VHL or the components of the E3 ubiquitin ligase complex. These results demonstrate that HAUSP decreases HIF-1α ubiquitination through recognizing a consensus sequence located in HIF-1α.

### HAUSP stabilizes HIF-1α to promote EMT and metastasis

To substantiate the *in vivo* stabilization of HIF-1α by HAUSP, a transient co-transfection experiment showed that overexpression of HAUSP increased HIF-1α at the protein levels but not the mRNA levels (Fig. 1f and Supplementary Fig. 1m), and further activated a *Twist1* promoter-driven reporter construct (Fig. 1g)^38^. To investigate the *in vivo* function of HAUSP, knockdown of HAUSP in H1299 cells showed a decrease in endogenous HIF-1α protein levels with no alteration in HIF-1α mRNA, which caused a reduction in Twist1 mRNA and protein levels (Fig. 1h and Supplementary Fig. 1n). Other HIF-1α downstream targets (*VEGF* and *Glut1*)^1^ were also repressed through HAUSP knockdown (Fig. 1h). Together, these gene expression changes led to a reversed EMT^5^,^6^, decreased *in vitro* migration/invasion and reduced *in vivo* metastatic activity of H1299 cells (Fig. 1h–l). Similar results were observed when knockdown of HAUSP in two other cell lines, SAS and MDA-MB-231, were performed (Supplementary Fig. 1o,p). In corroboration of the above finding, the prostate cancer cell line (PC3) was grown under hypoxia to induce the EMT. Knockdown of HAUSP in PC3 cells lowered the endogenous HIF-1α levels, but not the endogenous HIF-1α mRNA levels, induced by hypoxia and abolished hypoxia-triggered EMT and the *in vitro* migration and invasion activities (Supplementary Fig. 1q–t). These results indicate that HAUSP plays an essential role in the stabilization of HIF-1α induced by hypoxia, thereby activating HIF-1α target gene expressions and promoting the EMT and metastatic activities of cancer cells.

### Hypoxia induces K63-linked polyubiquitination of HAUSP

**Figure 2:**
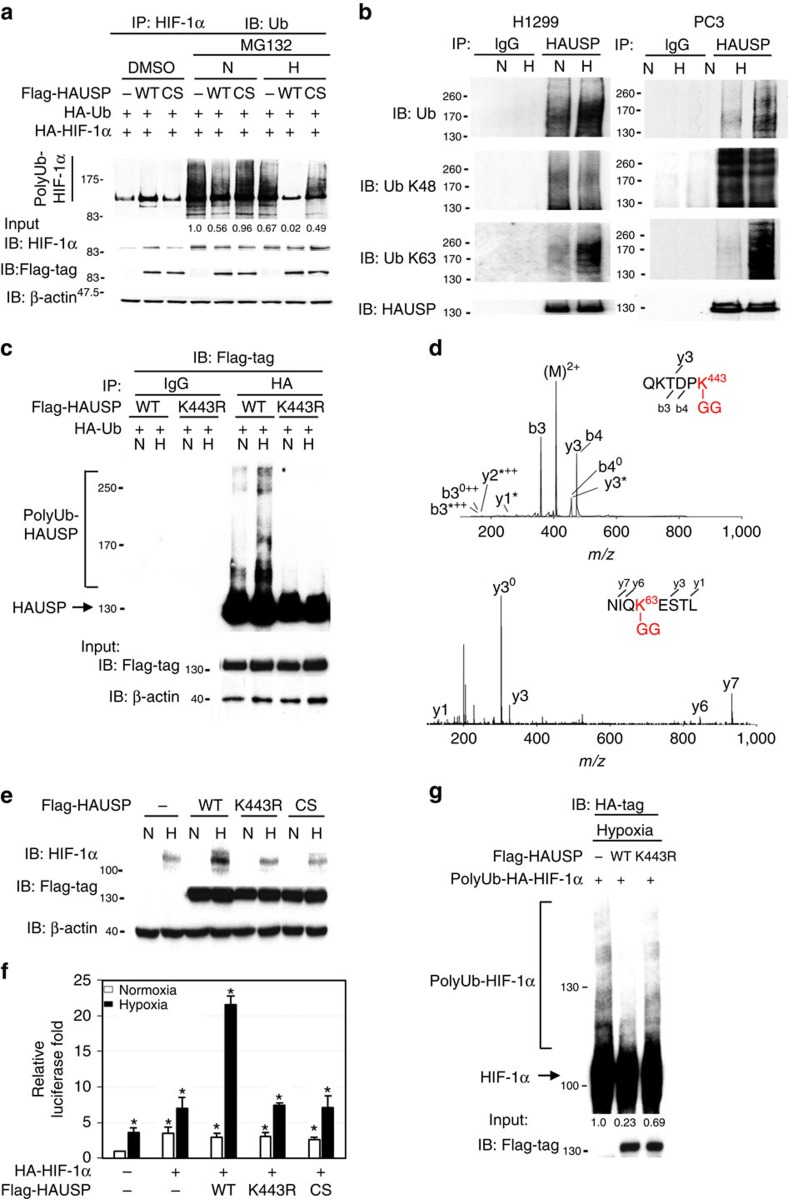
Increased K63-linked polyubiquitination of HAUSP at amino acid K443 under hypoxia and its identification by MS/MS spectra. (**a**) Hypoxia further increased the deubiquitinase activity of HAUSP when HAUSP (wild type or CS mutant), HIF-1α, HA-ubiquitin were co-expressed in 293T cells under MG-132 treatment to measure the polyubiquitinated HIF-1α levels under normoxia (N) or hypoxia (H). The densitometer reading of polyubiquitinated HIF-1α levels was listed underneath each lane. The first lane was arbitrarily set as 1. (**b**) Increased K63-linked polyubiquitination of HAUSP under hypoxia in two cell lines (H1299 and PC3) using anti-HAUSP antibodies to pull down HAUSP proteins followed by Western blot analysis using either anti-K63, anti-K48 or anti-ubiquitin antibodies. N, normoxia; H, hypoxia. (**c**) Increased polyubiquitination in HAUSP wild type, but not the K443R mutant, under hypoxia when HAUSP (wild type or K443R mutant) and HA-ubiquitin were co-expressed in 293T cells followed by precipitation by anti-HA antibodies. (**d**) The ubiquitination site K443 on QKTDPK (2+, *m/z* 416.2) was annotated to conjugate with ubiquitin-remnant peptide, Gly-Gly adduct. The peptide NIQKESTL (2+, *m/z* 524.2) of ubiquitin containing Gly-Gly adduct was identified from digested HAUSP in hypoxia. (**e**) Inability of the HAUSP^K443R^ mutant to increase the HIF-1α levels when they were co-expressed in 293T cells by Western blot analysis. The HAUSP-CS mutant was used as a negative control. (**f**) Co-expression of wild type, but not the K443R mutant, HAUSP with HIF-1α further increased the *Twist1* promoter-driven reporter activity in transient transfection assays. The condition not-transfected with HIF-1α or HAUSP plasmids under normoxia was used as a control. Data from three independent experiments are expressed as mean±s.d. The asterisk (*) indicated statistical significance (*P*<0.05) between experimental and control transfections, *t*-test. (**g**) *In vitro* deubiquitination assays showed that the HAUSP wild type (WT), but not the K443R mutant, deubiquitinated the polyubiquitinated HIF-1α. The densitometer reading of polyubiquitinated HIF-1α levels was listed underneath each lane. The first lane was arbitrarily set as 1.

Although overexpression of HAUSP is able to deubiquitinate HIF-1α under normoxia, we tested whether hypoxia affects the activity of HAUSP. Interestingly, HAUSP had a higher deubiquitinase activity under hypoxia (Fig. 2a). Since protein ubiquitination may modulate the activity and localization of proteins and HAUSP is polyubiquitinated as previously reported^23^,^39^, whether HAUSP could be ubiquitinated under hypoxia was tested. Using anti-K48-linked and anti-K63-linked polyubiquitination antibodies, the K63-linked polyubiquitination levels of HAUSP were increased under hypoxia compared to that through K48 (Fig. 2b). We further mapped HAUSP regions where ubiquitin moieties were linked through K63 using various truncation mutants of HAUSP together with wild-type or point mutants of ubiquitin (K48R or K63R ubiquitin). The results showed that K63-linked polyubiquitination occurred within the a.a. 400–450 region in HAUSP (Supplementary Fig. 2a,b). Further mutation analysis revealed that truncated HAUSP-K443R lost ubiquitin assembly under hypoxia (Supplementary Fig. 2c), as confirmed by overexpressing the full-length HAUSP wild-type or K443R in 293T cells under normoxia or hypoxia (Fig. 2c). Only K48R-ubiquitin was ligated to wild-type HAUSP, but not to the HAUSP^K443R^ mutant, under hypoxia (Supplementary Fig. 2d). On the contrary, the K63R-ubiquitin mutant was not able to form the ubiquitin chains on either HAUSP wild-type or HAUSP^K443R^ mutant (Supplementary Fig. 2e). To confirm the ubiquitination site and the ubiquitin linkage on HAUSP proteins, we further used mass spectrometry to directly identify the ubiquitination on K443 of HAUSP. The MS/MS spectrum provided the direct evidence to show that the ubiquitin-remnant peptide, Gly-Gly adduct, was exhibited on the ɛ-amine of K443 (Fig. 2d, upper panel). Moreover, the ubiquitin peptide containing K63 was also identified to contain the linkage of Gly-Gly adduct (Fig. 2d, lower panel). The result indicated that ubiquitination was modified on K443 of HAUSP protein and its ubiquitination was K63-linked. To further test the functional significance of K443 ubiquitination of HAUSP, the HAUSP^K443R^ mutant could not increase the HIF-1α levels or further increase the reporter construct activity activated by HIF-1α under hypoxia (Fig. 2e,f). *In vivo* deubiquitination assays showed that only wild-type HAUSP significantly decreased the polyubiquitinated HIF-1α levels (Supplementary Fig. 2f). *In vitro* deubiquitination assays using purified polyubiquitinated HIF-1α and Flag-HAUSP (wild-type versus K443R mutant) showed that the wild-type HAUSP, but not the K443R mutant, significantly deubiquitinated the polyubiquitinated HIF-1α species (Fig. 2g). The K63-polyubiquitinated HAUSP species were mainly located in the nucleus and increased under hypoxia (Supplementary Fig. 2g). Further co-immunoprecipitation experiments showed that the interaction between HAUSP^K443R^ and HIF-1α in hypoxia was significantly decreased compared to wild-type HAUSP (Supplementary Fig. 2h). These results indicate that polyubiquitination at K443 position of HAUSP is crucial for HAUSP to interact with its substrates. To determine whether hypoxia induces K63-linked polyubiquitination of proteins, we observed the presence of K63-linked polyubiquitination of CHK2 and NBS1 under hypoxia (Supplementary Fig. 2i), indicating that K63-linked polyubiquitination of proteins under hypoxia may be a general phenomenon.

### K63-polyubiquitinated HAUSP induces H3K56 acetylation

**Figure 3:**
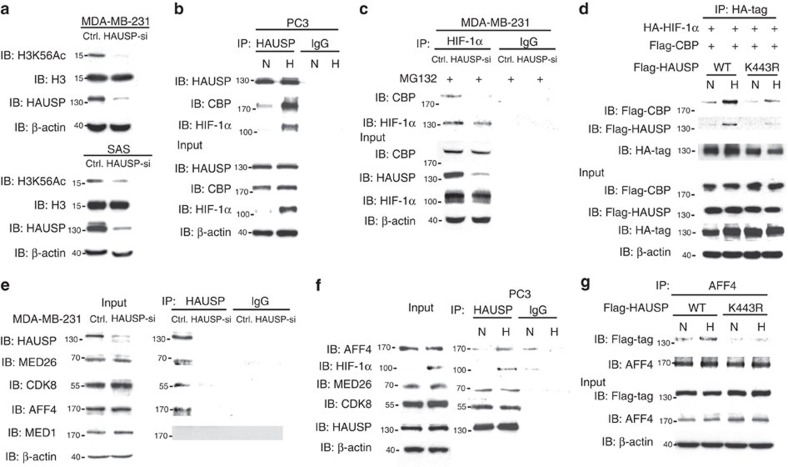
Polyubiquitinated HAUSP regulates global H3K56Ac and interacts with CBP and components of the Mediator/elongation complex. (**a**) Knockdown of HAUSP in MDA-MB-231 and SAS cells decreased the global levels of H3K56Ac by Western blot analysis. (**b**) Co-immunoprecipitation assays showed the increased interaction between HAUSP and CBP under hypoxia in PC3 cells. (**c**) Knockdown of HAUSP in MDA-MB-231 cells decreased the interaction between HIF-1α and CBP by co-immunoprecipitation assays. (**d**) Co-immunoprecipitation assays showed that the K443R HAUSP mutant had a weaker interaction with HIF-1α and CBP under hypoxia compared to wild-type HAUSP when the indicated proteins were expressed in 293T cells. (**e**) Co-immunoprecipitation assays showed the interaction between HAUSP and the components of the Mediator complex in MDA-MB-231 cells. (**f**) Increased interaction between HAUSP and AFF4 in PC3 cells under hypoxia by co-immunoprecipitation assays. (**g**) The HAUSP^K443R^ mutant had a decreased interaction with AFF4 compared to the wild-type HAUSP by co-immunoprecipitation assays when HAUSP (wild type or mutant) was overexpressed in 293T cells to pull down AFF4.

Since K63-linked polyubiquitin chains may serve as a scaffold protein to assemble a protein complex^27^ and there is an interaction between a deubiquitinase and chromatin complex^27^,^40^, we tested whether knockdown of HAUSP could disassemble a chromatin complex followed by a decrease in the global expression of histone marks. Screening of various histone marks showed that knockdown of HAUSP decreased the H3K56Ac levels in two cell lines (Fig. 3a), whereas other histone marks, including H3K4Ac, H3K14Ac, H3K18Ac, H3K27Ac, H2AK5Ac, H2A.Z and H4K12Ac, were unaffected (Supplementary Fig. 3a). Previous reports showed that CBP/p300 mediates H3K56 acetylation and serves as a co-activator of HIF-1α^34^,^41^. Further experiments showed that HAUSP interacted with CBP under normoxia and there was an increased interaction between HAUSP and CBP together with HIF-1α under hypoxia (Fig. 3b and Supplementary Fig. 3b). The increased interaction between HAUSP and CBP was mainly located in the nucleus under hypoxia (Supplementary Fig. 3c). We performed co-immunoprecipitation experiments using anti-HIF-1α antibodies to determine the interaction between HIF-1α and CBP in control versus HAUSP knockdown cells under MG132 treatment. Less amount of CBP was associated with HIF-1α in HAUSP knockdown cells (Fig. 3c and Supplementary Fig. 3d). Overexpressing either the wild-type HAUSP or K443R mutant followed by the pull-down assay using anti-CBP antibodies showed that the interaction between HAUSP^K443R^ and CBP was decreased compared to the interaction between HAUSP and CBP under hypoxia (Supplementary Fig. 3e). We further examined whether Lysine 443 of HAUSP is essential to enhance interaction between HAUSP, CBP and HIF-1α. The wild-type HAUSP or K443R mutant were co-expressed with HIF-1α and CBP to perform co-immunoprecipitation experiments by the pull-down assay using anti-HIF-1α antibodies. The result showed that the interaction between HIF-1α and CBP was significantly decreased when HAUSP^K443R^ was expressed compared to the wild-type HAUSP, indicating that HAUSP may serve as a scaffold to anchor the formation of the HIF-1α-CBP transcription complex and the Lysine 443 residue is required for the interaction under hypoxia (Fig. 3d).

We also tested the ability of HAUSP to anchor the formation of CDK8-mediator complex and the result showed that HAUSP interacted with CDK8 and other components of the Mediator complex including the core Mediator subunit (MED26) and the scaffold protein of super elongation complex (SEC), AFF4 (refs 30, 42, 43), but not with another component of the Mediator complex, MED1 (Fig. 3e and Supplementary Fig. 3f). HAUSP did not interact with H2A.Z or Pol II (Supplementary Fig. 3g). Further experiments showed that the interaction between HAUSP and AFF4 was increased under hypoxia, but not between HAUSP and CDK8/MED26 (Fig. 3f and Supplementary Fig. 3h), and HAUSP^K443R^ had a significantly reduced interaction with AFF4 under hypoxia compared to HAUSP (Fig. 3g).

### qChIP analysis of proteins and histone marks at promoters

**Figure 4:**
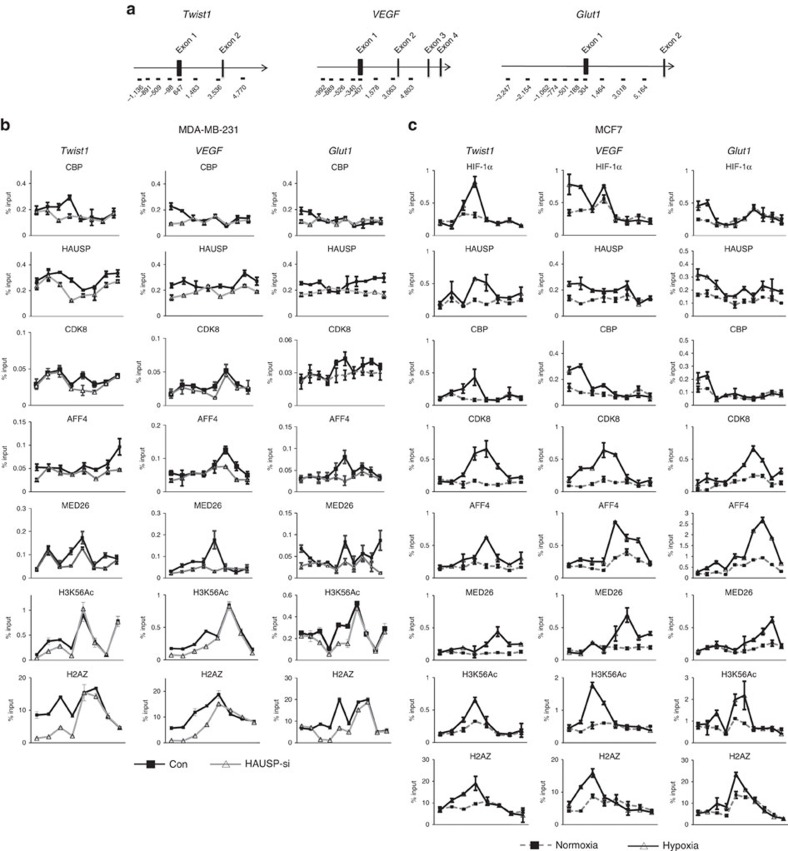
qChIP assays of the binding of various proteins as well as the levels of H3K56Ac and H2A.Z on the promoters of HIF-1α target genes. (**a**) The genomic regions of *Twist1, VEGF* and *Glut1* genes and the regions that were checked by qChIP assays. (**b**) qChIP levels of the binding of various proteins and histone marks on the promoters/gene body of three genes (*Twist1, VEGF, Glut1*) between control and HAUSP knockdown clone in MDA-MB-231 cells. Different regions located in the promoters and inside the gene body of three genes were shown underneath the plotting of genes. The different numbers indicated the starting nucleotide positions in the regions (labelled by brackets) before and after the initiation site. (**c**) qChIP levels of the binding of various proteins and histone marks on the promoters/gene body of three genes (*Twist1, VEGF, Glut1*) between normoxia and hypoxia in MCF7 cells. Different regions located in the promoters and inside the gene body of three genes were shown underneath the plotting of genes. The different numbers indicated the starting nucleotide positions in the regions (labelled by brackets) before and after the initiation site. For the qChIP analysis in (**b**,**c**), data from three independent experiments are expressed as mean±s.d.

Next, we determined the correlation between the presence of H3K56Ac histone mark and the expression of HIF-1α downstream target genes. Quantitative chromatin immunoprecipitation (qChIP) assays were performed to examine the presence of H3K56Ac at the promoters of HIF-1α target genes (Fig. 4a), including *Twist1*, *VEGF* and *Glut1* in two cell lines (MDA-MB-231, SAS). As anticipated, the levels of H3K56Ac decreased at the promoters of these three genes in HAUSP knockdown cells (Fig. 4b and Supplementary Fig. 4a). We also examined the H2A.Z levels since H3K56Ac mediates the turnover of a histone variant H2A.Z and the presence of both indicates the promoter with high nucleosome turnover and active transcription^44^,^45^. Knockdown of HAUSP resulted in a decreased H2A.Z level at the promoters of these three genes (Fig. 4b and Supplementary Fig. 4a). The levels of CBP binding on the promoters of these three genes were decreased under HAUSP knockdown (Fig. 4b and Supplementary Fig. 4a). In contrast, the levels of AFF4 and MED26 were mainly located in the gene body, in support of their roles in transcription elongation (Fig. 4b and Supplementary Fig. 4a). Finally, knockdown of HAUSP decreased the binding of HAUSP in both the promoter region and gene body of these genes, indicating the dual role of HAUSP in maintaining H3K56Ac mark and interacting with co-activators and the components of Mediator/SEC (Fig. 4b and Supplementary Fig. 4a). There was no difference in the H3K56Ac levels on the promoters of *E-cadherin*, *N-cadherin, actin* and *GAPDH* under HAUSP knockdown (Supplementary Fig. 4b). To further confirm the levels of histone marks and factors binding on the promoters of these HIF-1α target genes, two cell lines (PC3, MCF7) under hypoxia were tested by qChIP assays. The results showed that the binding of HIF-1α, CBP, H3K56Ac and H2A.Z was limited to the promoter regions; whereas the binding of AFF4 and MED26 was mostly in gene body (Fig. 4c and Supplementary Fig. 4c), consistent with the HAUSP knockdown results (Fig. 4b and Supplementary Fig. 4a). The binding of CDK8 was shown in the promoter regions and gene body, which coincided with the presence of HAUSP binding (Fig. 4b,c and Supplementary Fig. 4a,c). HAUSP also bound to the promoters of other HIF-1α target genes (*LDHA*, *TFRC* and *RARA*) by qChIP analysis, whereas knockdown of HAUSP significantly decreased the binding of HAUSP and the H3K56Ac levels on their promoters (Supplementary Fig. 4d). The above analyses suggest that HAUSP and its polyubiquitinated form anchor HIF-1α, CBP, and the components of the Mediator/SEC (CDK8, AFF4), acting as a master scaffold and mediating transcription initiation and elongation of HIF-1α target genes.

### CBP is a ubiquitin receptor for K63-polyubiquitinated HAUSP

**Figure 5:**
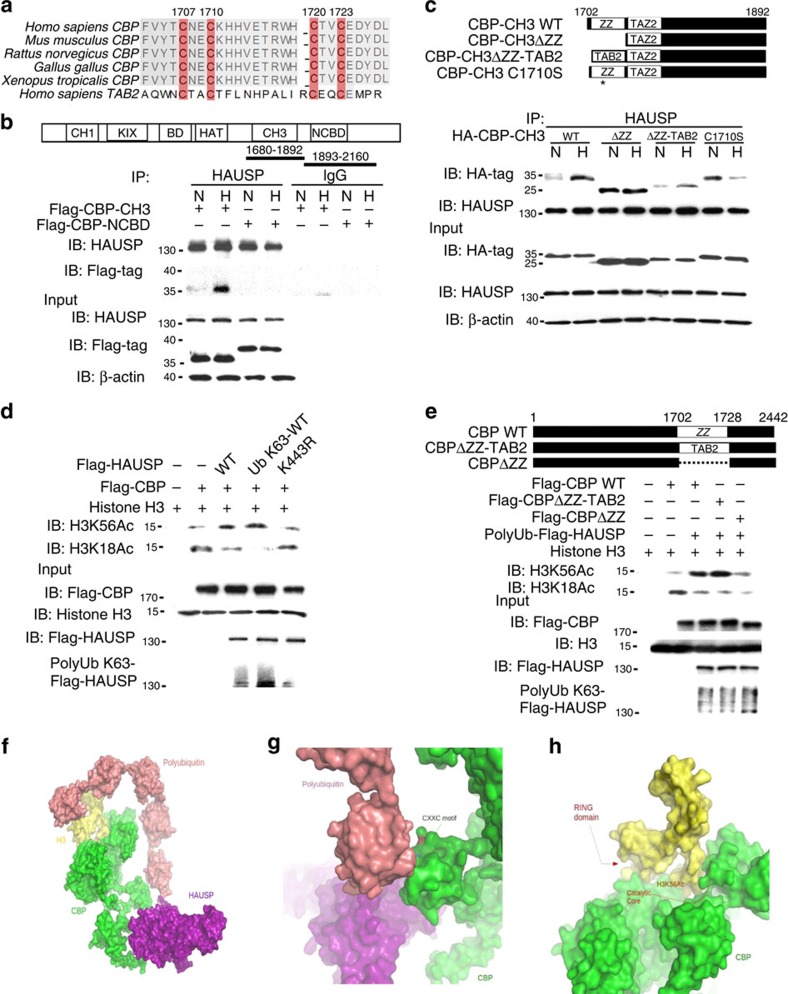
Interaction of HAUSP with a ubiquitin receptor (CBP) and a simulation model of the CBP-polyubiquitinated HAUSP-histone 3 complex. (**a**) The homology comparison between the ZZ-type zinc finger motifs of CBP from different species and the zinc finger motifs of human TAB2. (**b**) Co-immunoprecipitation assays showed that the increased interaction between the CBP CH3 domain and HAUSP under hypoxia when the indicated proteins were overexpressed in 293T cells. (**c**) The upper panel shows the schematic diagram of CBP and different CBP mutants. The lower panel shows the increased interaction between the CBP CH3 domain/TAB2 zinc finger motifs substituted CBP and HAUSP under hypoxia by co-immunoprecipitation assays in 293T cells overexpressing these indicated proteins. There was no increased interaction between the CBP CH3 domain-ZZ-type zinc finger motif deleted/mutated and HAUSP under hypoxia. (**d**) *In vitro* histone acetylation assays showed that the H3K56 acetylation levels were maximally induced by K63-polyubiquitinated HAUSP compared to the HAUSP wild type or HAUSP^K443R^ mutant, whereas H3K18 acetylation was drastically decreased in the presence of K63-polyubiquitinated HAUSP. (**e**) The upper panel shows the schematic diagram of CBP and different CBP mutants. The lower panel shows that the CBP wild type and CBP with the ZZ-type zinc finger motif substituted by Zinc-finger of TAB2 increased the H3K56Ac levels in the presence of K63-polyubiquitinated HAUSP, whereas the CBP mutant with deleted ZZ-type zinc finger motif did not increase the H3K56Ac levels. The H3K18Ac levels were used as controls. (**f**) A hypothetical model of three-dimensional macromolecular structures and complexes involving K63-polyubiquitined HAUSP, CBP and H3. (**g**) An enlarged view of the hypothetical model showed the interaction between CXXC motif of CBP and the polyubquitin chain. (**h**) An enlarged view of the hypothetical model showed that the region surrounding H3K56 entered into the catalytic core between the HAT and RING domains of CBP. The RING domain is located in the a.a. 1204–1278 region of CBP. Protein surface models were used to depict the models in **f**–**h**.

Since HAUSP is K63-polyubiquitinated under hypoxia, we searched for the ubiquitin receptor of this K63 polyubiquitin chain. We first showed the enhancement of the histone acetyltransferase activity of CBP in cells overexpressing HAUSP, but not HAUSP^K443R^, as the H3K56Ac levels were significantly increased (Supplementary Fig. 5a). The ZZ-type zinc finger (ZZ) domain of CBP is highly similar to zinc finger (ZnF) domain of TAB2, the ubiquitin receptor for K63-polyubiquitinated TAK1 (refs 46, 47) (Fig. 5a). To test whether the ZZ-type zinc finger motif (ZZ) of CBP is a ubiquitin receptor for the K63 polyubiquitin chain, co-immunoprecipitaion experiments showed that CH3 domain of CBP that contains the putative ZZ-type zinc finger motif (a.a. 1680–1892) interacted with HAUSP, but not the NCBD domain containing nuclear co-activator binding domain (a.a. 1893 to 2160) (Supplementary Fig. 5b). Further domain mapping experiments showed that the a.a. 200–300 domain of HAUSP interacted with the CH3 domain in CBP (Supplementary Fig. 5c). In addition, there is an increased interaction of the CH3 domain in HAUSP under hypoxia (Fig. 5b). We further tested whether the ZZ motif in CBP functions similar to the ZnF motif in TAB2. We made a construct containing the deleted ZZ motif in the CH3 domain (ΔZZ) and substituted it with the similar motif from TAB2 (ΔZZ-TAB2), and another construct containing a mutated C in the ZZ motif (C1710S) to perform co-IP experiments. The results showed that both the wild-type CH3 domain and the TAB2 zinc finger-substituted CH3 domain (ΔZZ-TAB2) had an increased interaction with HAUSP under hypoxia, but not the zinc finger deleted (ΔZnF) or mutated (C1710S) mutant (Fig. 5c). These results demonstrated that CBP is a ubiquitin receptor for K63-polyubiquitinated HAUSP.

Since knockdown of HAUSP only abolished the global H3K56Ac levels (Fig. 3a), we tested whether HAUSP and its K63-polyubiquitinated form could regulate the histone substrate selectivity mediated by CBP since CBP acetylates various lysine residues on histone 3 (for example, H3K18, H3K56, and so on) (refs 34, 36). We first tested whether the CBP-K63 polyubiquitinated HASUP complex had a higher binding affinity to histone 3 versus histone 4. The results showed that anti-histone 3 antibodies, but not anti-histone 4 antibodies, pulled down the most amount of the CBP-K63 polyubiquitinated HAUSP complex (Supplementary Fig. 5d), indicating a preferential binding of the CBP-polyubiquitinated HAUSP complex to histone 3. The result was confirmed using anti-HAUSP antibodies to pull down more histone 3 under hypoxia (Supplementary Fig. 5e), indicating that K63-polyubiquitinated HAUSP was more chromatin bound under hypoxia. This substrate selectivity was further confirmed using an *in vitro* histone acetylation assay. The highest H3K56Ac levels were detected by anti-H3K56Ac antibodies in the presence of wild-type K63-polyubiquitinated HAUSP (Fig. 5d). However, incubation with HAUSP decreased the H3K18Ac levels and incubation with K63-polyubiquitinated HAUSP further decreased the H3K18Ac levels (Fig. 5d), indicating that K63 polyubiquitinated HAUSP not only enhanced the enzymatic activity, but also determined the substrate specificity of CBP. To further demonstrate that CBP is a ubiquitin receptor for K63-polyubiquitinated HASUP, we made several CBP mutant constructs, including the ZZ motif replaced by Znf motif in TAB2 (ΔZZ-TAB2) and the truncated ZZ motif in CBP (ΔZZ, as a negative control). The *in vitro* histone acetylation assays showed that the H3K56Ac levels were increased by the CBP WT or ΔZZ-TAB2 mutant-K63-polyubiquitinated HAUSP complex, but not by the CBPΔZZ mutant-K63-polyubiquitinated HAUSP complex (Fig. 5e). In addition, the H3K18Ac levels were also decreased by the CBP WT or ΔZZ-TAB2-K63-polyubiquitinated HAUSP complex (Fig. 5e). These results showed that both the ZZ motif in CBP and K63-polyubiquitinated HAUSP are crucial for H3K56 acetylation mediated by CBP. The computer model drawn by PyMol was used to explain our speculated inter-molecule interactions, which showed that polyubiquitinated HAUSP interacted with CBP to change its conformation and the K63 polyubiquitin chains positioned CBP to specifically recognize the H3K56 site (Fig. 5f). After the interaction between CBP and K63-polyubiquitinated HAUSP (Fig. 5g), the RING domain in CBP moved away from the catalytic core and caused the exposure of catalytic core to accommodate the H3K56 substrate, but not the H3K18 substrate (Fig. 5g,h), explaining the possible substrate selectivity of the CBP-polyubiquitinated HAUSP complex to acetylate H3K56.

### ChIP-seq analysis of HAUSP and HIF-1α targets

**Figure 6:**
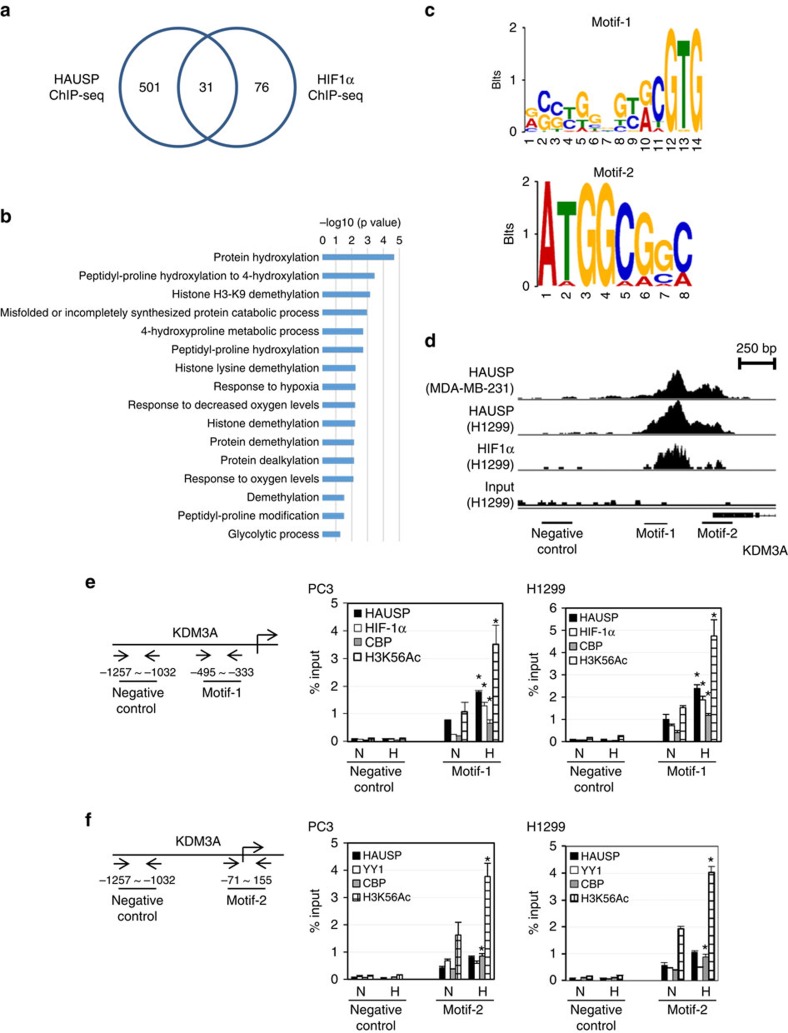
Analysis of ChIP-seq results with identification of two motifs and confirmation of various factors binding to the *KDM3A* promoter regions. (**a**) The numbers of overlapping set of genes that were bound by HAUSP and HIF-1α by ChIP-seq analysis. (**b**) GO analysis of the 31 genes that showed binding by both HAUSP and HIF-1α. (**c**) Identification of two consensus motifs that were bound by HAUSP and HIF-1α. (**d**) Gene track analysis of a representative gene, *KDM3A*, that contained motif-1 and motif-2 in its promoter regions. (**e**) The left panel shows the schematic diagram of the *KDM3A* promoter regions containing motif-1 that were checked by qChIP assays. qChIP analysis confirmed the binding of various factors to motif-1 in the *KDM3A* promoter regions. (**f**) The left panel shows the schematic diagram of the *KDM3A* promoter regions containing motif-2 that were checked by qChIP assays. qChIP analysis confirmed the binding of various factors to motif-2 in the *KDM3A* promoter regions. N, normoxia; H, hypoxia. Data from three independent experiments are expressed as mean±s.d. The asterisk (*) indicated statistical significance (*P*<0.05) between normoxia and hypoxia conditions, *t*-test.

To explore the chromatin modification dynamics regulated by HAUSP in the HIF-1α target genes, we next screened for the genes whose promoters were bound by HAUSP using ChIP-seq analysis. The distribution of HAUSP binding peaks were centred around the transcription start sites (TSS)(Supplementary Fig. 6a). Five hundred thirty-two genes contained the HAUSP binding peaks and Gene Ontology (GO) analysis showed that they belonged to various biological processes (Supplementary Fig. 6b). We again performed ChIP-seq analysis of HIF-1α binding. Further analysis showed that there were 31 genes containing peaks in their promoters bound by HIF-1α and HAUSP and GO analysis of these genes was shown (Fig. 6a,b). We searched for the consensus motifs in the peaks bound by HIF-1α and HAUSP and two different kinds of motifs were discovered (Fig. 6c). Interestingly, motif-1 contained the HIF-1α binding consensus sequence (ACGTG), whereas motif-2 was highly homologous to the YY1 binding site (Fig. 6c). We also performed a similar analysis using the HIF-1α ChIP-seq results from another data base and it overlapped with our HAUSP ChIP-seq results (Supplementary Fig. 6c). Between these two sets of overlapping genes, both motif-1 and motif-2 could also be discovered (Supplementary Data 1). The HIF-1α and HAUSP ChIP-seq gene tracks of a representative gene, lysine demethylase 3A (*KDM3A*), were shown, which contained the positions of motif-1 and motif-2 (Fig. 6d). qChIP analysis was used to examine the binding of HIF-1α, CBP, HAUSP and the H3K56Ac levels at the *KDM3A* promoter. While the binding of HIF-1α, CBP and HAUSP to the *KDM3A* promoter was detected under normoxia, there was increased binding of all these three factors to motif-1 in the *KDM3A* promoter under hypoxia, leading to the increased H3K56Ac levels on the promoter (Fig. 6e). However, the binding of HAUSP and YY1 to motif-2 was not increased under hypoxia. In contrast, increased H3K56Ac levels were observed in motif-2 under hypoxia, indicating that K63-polyubiquitinated HAUSP enhanced the ability of CBP to acetylate H3K56 (Fig. 6f). There was no motif-1 or motif-2 present at the promoters of *E-cadherin* and *N-cadherin* and there was no difference in the binding of HAUSP or the H3K56Ac levels in these promoters under HAUSP knockdown (Supplementary Fig. 4b). We also observed similar qChIP results in the promoters of *Twist1*, *VEGF* and *Glut1* genes, which contained motif-1 and motif-2 in their promoters (Supplementary Fig. 6d,e). These results indicate that the regulation of hypoxia-induced gene expression is mediated through a unique mechanism when HIF-1α is assisted by HAUSP to induce its target gene expression.

### Characterization of Hausp^K444R^ mouse fibroblasts

**Figure 7:**
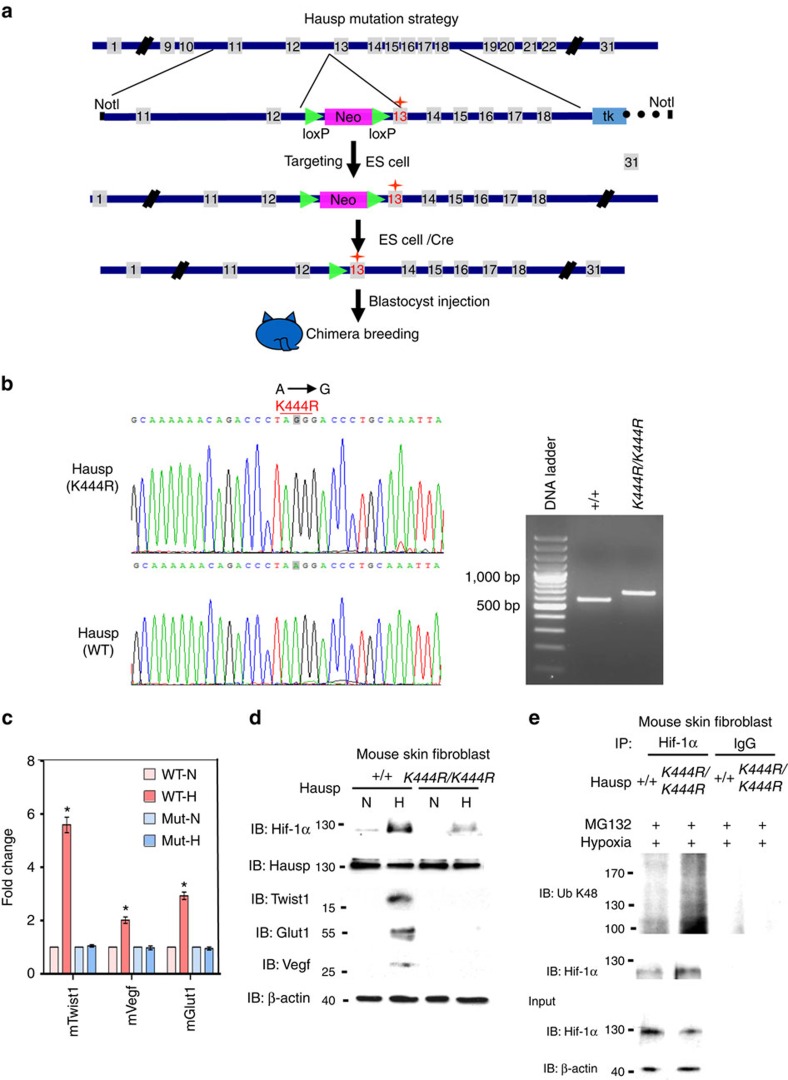
Hausp point mutation strategy in knock-in mouse strain and its identification through characterization of hypoxia response in fibroblasts. (**a**) The point mutation strategy used to generate Hausp K444R point mutant in mouse ES cells. (**b**) Identification of Hausp K444R mutation (A to G) in mouse skin fibroblast cells by sequencing and PCR analysis. cDNA products from wild-type (Hausp^+/+^) and from K444R homozygous (HAUSP^K444R/K444R^) were amplified by PCR using HAUSP typing primers to generate the 594 bp (WT) and 694 bp (K444R) products. (**c**) Real-time PCR analysis to examine the response of three mouse Hif-1α target gene expression under hypoxia. WT, wild type; Mut, point mutant; N, normoxia; H, hypoxia. Data from three independent experiments are expressed as mean±s.d. The asterisk (*) indicates statistical significance (*P*<0.05) between experimental and control cells, *t*-test. (**d**) Western blot analysis showed that the expressions of Hif-1α downstream targets were decreased in Hausp K444R mutant mouse skin fibroblasts under hypoxia. (**e**) Western blot analysis showed that the K48 polyubiquitination levels of Hif-1α were increased in the Hausp homozygous K444R mouse skin fibroblasts treated with MG132 under hypoxia compared to those in the wild-type Hausp mouse skin fibroblasts.

In order to test the *in vivo* effect of K443R mutation in HAUSP, we generated a homozygous Hausp K444R point mutant (corresponding to K443R mutation in HAUSP) mouse strain by a conventional knock-in approach (Fig. 7a). The K444R point mutant mouse strain was confirmed by sequencing and PCR analysis (Fig. 7b). The skin fibroblasts from wild-type Hausp and K444R mutant mice were isolated and tested under hypoxia. Real-time PCR assays showed that the response of *mTwsit1*, *Vegf* and *mGlut1* genes to hypoxia was abolished in Hausp^K444R^ mouse fibroblasts (Fig. 7b). Western blot analysis showed that mouse Twist1, Vegf and Glut1 proteins were not induced by hypoxia in Hausp^K444R^ fibroblasts (Fig. 7d). Wild-type Hausp, but not Hausp^K444R^, decreased the K48-linked polyubiquitination levels of Hif-1α in mouse fibroblasts under MG132 treatment (Fig. 7e). A baseline level of Hif-1α was also expressed in the mouse skin fibroblast cells, which interacted with Hausp under normoxia (Supplementary Fig. 6f). The above results confirmed our *in vitro* observations.

### HectH9 is the E3 ligase for HAUSP under hypoxia

**Figure 8:**
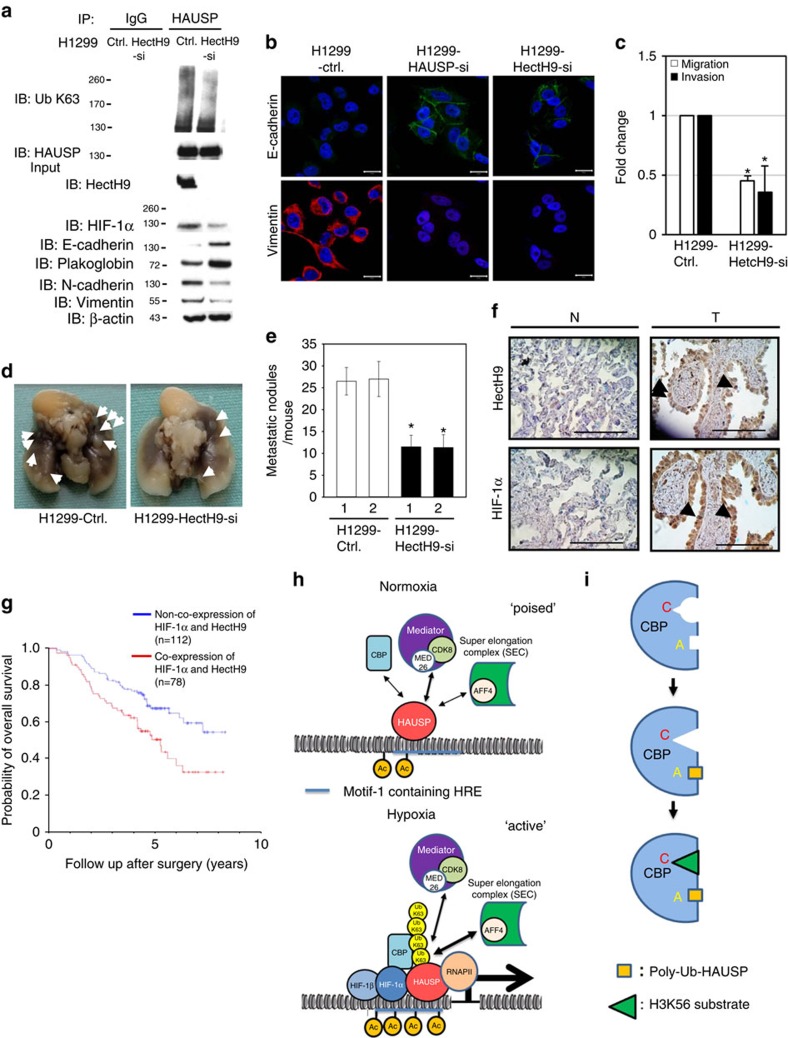
HectH9 knockdown reverses EMT and decreases metastatic activity and its prognostic value with HIF-1α and models. (**a**) Knockdown of HectH9 in H1299 cells decreased the levels of K63-polyubiquitinated HAUSP, reversed EMT, and decreased the expression of HIF-1α targets. (**b**) Immunofluorescence staining of E-cadherin and vimentin in H1299 cells (control versus HectH9 knockdown). (**c**–**e**) Knockdown of HectH9 decreased the *in vitro* migration/invasion and *in vivo* metastatic activity of H1299 cells. For (**c**), data from three independent experiments are expressed as mean±s.d. For (**e**), data are presented as the mean±s.d. (*n*=6). The asterisk (*) indicated statistical significance (*P*<0.05) between HectH9 knockdown and control knockdown, *t*-test. (**f**) Immunohistochemical staining of co-expression of HectH9 and HIF-1α in corresponding normal tissue (N) and primary tumour (T) of a representative lung adenocarcinoma patient case. The samples prepared for co-expression analysis were cut and examined at the same region. Black arrows indicate the nuclear expression of HectH9 and HIF-1α. Photographs were taken at magnifications of × 400. Scale bars represent 100 mm. (**g**) Cumulative probability of overall survival stratified by co-overexpression of HIF-1α and HectH9 in lung cancer patients. Statistical significance was shown between co-expression of HIF-1α/HectH9 and non-co-expression patient groups. (**h**) A model to depict the role of HAUSP in two different status (normoxia-poised versus hypoxia-active). HAUSP has a weak interaction with CBP and components of the Mediator/elongation complex in a ‘poised' status under normoxia. Under hypoxia, there is increased interaction between K63-polyubiquitinated HAUSP and HIF-1α/CBP/AFF4 in an ‘active' status to mediate HIF-1α-induced gene transcription. This multi-protein complex also induces H3K56Ac through the enzyme-substrate plasticity induced by K63-polyubiquitinated HAUSP. (**i**) A model to depict the role of K63-polyubqiutinated HAUSP to allosterically modulate CBP so CBP can only acetylate H3K56 site. The triangle represents the H3K56 substrate, in contrast to the circle that represents the H3K18 site.

To identify the E3 ubiquitin ligase that mediates the K63-linked polyubiquitination of HAUSP, the RNA levels of several K63-specific E3 ligases, including HectH9, TRAF6, RNF8, Siah2, cIAP1 and cIAP2, were screened to determine their induction by hypoxia^26^. Among them, only HectH9 and TRAF6 were induced by hypoxia (Supplementary Fig. 7a). Further experiments showed that knockdown of HectH9 significantly decreased the K63-polyubiquitination HAUSP levels compared to the control or TRAF6 knockdown (Supplementary Fig. 7b). Induction of HIF-1α levels by hypoxia was also significantly decreased in the HectH9 knockdown PC3 cells compared to the control knockdown cells (Supplementary Fig. 7b). The *in vitro* ubiquitination assay showed that HectH9 mediated K63-linked polyubiquitination of HAUSP, but not HAUSP^K443R^ (Supplementary Fig. 7c). Likewise, HectH9 knockdown in H1299, SAS, MDA-MB-231 cells abolished the K63-linked polyubiquitination of HAUSP and decreased the induction of HIF-1α levels under hypoxia (Fig. 8a and Supplementary Fig. 7d,e,j). Reversion of EMT (that is, restoration of E-cadherin/plakoglobin and repression of N-cadherin/vimentin) as well as repression of Twist1, VEGF and Glut1 expressions was detected in these HectH9-knockdown H1299, SAS and MDA-MB-231 cell models (Fig. 8a and Supplementary Fig. 7e,j). Immunofluorescence staining confirmed the reversion of EMT in these HectH9-knockdown cells (Fig. 8b and Supplementary Fig. 7f,k). Consistently, HectH9 knockdown resulted in the reduction of *in vitro* migration and invasion activities of H1299, SAS and MDA-MB-231 cells (Fig. 8c and Supplementary Fig. 7g,i,l). HectH9 knockdown also decreased the *in vivo* metastatic activity of H1299 and SAS cells (Fig. 8d,e and Supplementary Fig. 7h,i). These results indicate that HectH9 knockdown reduces the K63-polyubiquitinated HAUSP levels, decreases HIF-1α levels, reverses the EMT and reduces the metastatic activity of cancer cells under hypoxia.

### Co-expression of HectH9 and HIF-1α is a prognostic marker

To investigate whether HectH9-mediated HIF-1α stabilization indeed occurs in human cancers and to evaluate the prognostic significance of HIF-1α/HectH9 co-expression, tissue-microarray immunohistochemistry (IHC) analysis of HIF-1α and HectH9 expression was performed in 190 sets of resected stage I lung adenocarcinoma patient samples (Supplementary Table 1, IHC staining from a representative case was shown in Fig. 8f). Tumours with increased HIF-1α expression significantly correlated with HectH9 overexpression (*P*<0.001, *X*^2^ test, Supplementary Table 2). Prognostic prediction analysis showed that co-expression of HIF-1α and HectH9 had a significantly worse overall survival than non-co-expression cases (*P*=0.003, Kaplan-Meier Estimate, Fig. 8g). The prognostic effect of HIF-1α and HectH9 co-expression was independent of other prognostic markers (tumour size, T status) (*P*=0.001; multivariate analysis by Cox proportional hazards model, Supplementary Table 3). Collectively, the correlation analysis indicates that HectH9 expression correlates with HIF-1α levels and their association indeed occurs in lung adenocarcinoma samples. Survival analysis supports the prognostic value of co-expression of HIF-1α and HectH9 in lung cancer cases.

## Discussion

Deubiquitinases can function as oncogenes or tumour suppressors^48^. The role of HAUSP as a tumour suppressor or oncogene has been under debate. Recent results showed that HAUSP deubiquitinates PTEN to cause its nuclear exclusion and leads to tumour progression, supporting the oncogenic role of HAUSP^18^. Development of anti-HAUSP inhibitors to treat cancers also supports its oncogenic property^19^,^20^,^21^,^22^. In this report, we further demonstrate that the post-translational and epigenetic mechanisms mediated by K63-polyubiquitinated HAUSP are crucial for HAUSP to mediate hypoxia-induced EMT and metastasis. The blunted hypoxic response in the Hausp^K444R^ point mutant mouse fibroblasts further supports the importance of K63-linked polyubiquitination of HAUSP in response to hypoxic stress (Fig. 7). In addition to stabilizing HIF-1α through its deubiquitinase activity, polyubiquitinated HAUSP also dictates the CBP-substrate selectivity to specifically acetylate H3K56 on the promoter of HIF-1α target genes to facilitate hypoxic response. Our results provide a unique model of converting post-translational modification into epigenetic regulation and further implicate the role of HAUSP in the field of hypoxia-induced tumour progression since the other two deubiquitinases that could deubiquitinate HIF-1α, USP8 and UCHL1, are either involved in ciliogenesis under normoxia or does not modulate HIF-1α-induced gene transcription through regulating chromatin modification^49^,^50^.

Our ChIP-seq results identify two novel motifs that are responsive to hypoxia. Motif-1 contains the hypoxia-response element embedded in the motif. Motif-2 contains the YY1 consensus binding site. This discovery further defines the cis-acting elements that respond to hypoxia beyond the conventional hypoxia-response element described in literature. In addition, binding of HAUSP to chromatin under normoxia presents a ‘poised' status that will easily respond to hypoxia under an ‘active' transcription/chromatin modification status that more readily turn on the expression of HIF-1α target genes (a model is presented in Fig. 8h). K63-polyubiquitinated HAUSP may serve as a ‘master scaffold' to anchor the HIF-1α-CBP-Mediator-SEC complex. Another interesting observation is that the CBP-substrate selectivity can be achieved by K63-linked polyubiquitination of HAUSP, making the K63-polyubiquitinated HAUSP an allosteric modulator that will restrict CBP into recognizing H3K56, but not H3K18 (Fig. 5d,e). We propose that K63-polyubiquitinated HAUSP is a specific ‘histone mark specifier' for CBP. For this allosteric interaction, the ZZ motif in CBP becomes a type of ‘ubiquitin receptor' that was originally described in the NF-kB pathway^47^. In line with ubiquitin-mediated protein degradation or kinase activation^24^, this report is a good demonstration of ‘ubiquitin-mediated allosteric interaction and enzyme-substrate selectivity'. For allosteric interactions, the classical Monod-Wyman-Changeux model posits that enzyme activity can be allosterically modulated by its allosteric modulator^51^. Our results demonstrate that substrate selectivity can be modulated by an allosteric modulator (a model is shown in Fig. 8i), which expands the spectrum of allosteric interactions beyond the Monod-Wyman-Changeux model. For disease implications, the zinc finger domain in CBP is located outside of the HAT domain, supporting the occurrence of small gene deletions in Rubinstein-Taybi syndrome^52^. A missense mutation (C1710S) in CBP has been shown in a lung cancer case^53^. Structural relationship to delineate this observation is shown in Fig. 5f–h. These models represent highlights of K63-polyubiquitinated HAUSP to regulate specific histone modification and HIF-1α target gene expression in order to facilitate hypoxia-induced tumour progression.

Since both HIF-1α knockout and HAUSP knockout mice are embryonic lethal^54^,^55^, this report suggest that HAUSP knockout may develop embryonic lethality through the HIF-1α pathway. It is interesting that our Hausp^K444R^ point mutant knock-in mice survive well, but manifest physical anomaly. This mouse strain will be useful to study both the functions of HIF-1α and HAUSP since knockout of either gene presents an embryonic lethal phenotype. The role of HectH9 in serving as a prognostic marker together with HIF-1α further supports the oncogenic role of HAUSP since HAUSP is K63-polyubiquitinated by HectH9. The development of various anti-HAUSP drugs provides validation of HAUSP as a therapeutic target for the treatment of hypoxia-induced cancers/metastasis and other types of cancers including bortezomib-resistant multiple myeloma^19^,^20^,^21^,^22^.

## Methods

### Cell culture and oxygen deprivation

Human Lung cancer H1299, prostate cancer PC3, head and neck cancer SAS, embryonic kidney 293T, and breast cancer MDA-MB-231 cells were obtained from ATCC^38^,^56^. The cell lines showed negative results for Mycoplasma contamination. Oxygen deprivation was carried out in an incubator with 1% O_2_, 5% CO_2_ and 94% N_2_ for 18 h.

### Plasmids and stable transfection

The HAUSP and pHA-HIF-1α plasmids were obtained from Prof Wei Gu (Columbia University) and Dr L.E. Huang (University of Utah), respectively^38^,^56^. The oligonucleotides used to generate various plasmids and the methods to construct these plasmids were shown (Supplementary Tables 4 and 5). Calcium phosphate or Lipofectamine method was used to transfect plasmids. Briefly, plasmids were incubated with calcium phosphate or Lipofectamine reagent and further incubated with cells. After a set period, the cells were washed followed by antibiotics selection in one and a half days. All the stable clones were established by transfection of plasmids as designated by the name of the clones.

### Protein extraction and Western blot analysis

For extraction of proteins from cell lines, cell lysis buffer (50 mM Tris, pH 7.5, 30 mM MgSO_4_, 8 mM EDTA, 2 mM dithiothreitol (DTT) and 2% Triton X-100) containing protease inhibitors was used^38^,^56^. Cell lysates were clarified by centrifugation at 13,000 r.p.m., 4 °C for 10 min. The protein content was determined by Bradford method (Bio-Rad Laboratories, Hercules, CA, USA). For Western blot analysis, 50–100 μg protein extracts from each clone were loaded to 10% sodium dodecyl sulfate (SDS)-PAGE gels and transferred to nitrocellulose filters. The filters were probed with different antibodies, and an anti-ß-actin antibody was selected as a loading control. Signals were developed using an ECL chemiluminescence kit (Amersham Biosciences, UK). Molecular weight markers were labelled on the left side of each lane. The characteristics of the antibodies used were listed (Supplementary Table 6). The full Western blots of regular figures were shown in the Supplementary Information File (Supplementary Notes).

### RNA extraction and quantitative real-time PCR

Trizol (Invitrogen Life Technologies, Carlsbad, CA, USA) was used for RNA purification from cultured cells. Quantitative real-time PCR was done in a PRISM ABI7700 Sequence Detection System (Applied Biosystems, Foster City, CA, USA) with the preset PCR program, and TBP (TATA box binding protein) was selected as an internal control^38^,^56^. cDNA (0.1 μg) and primers (80 μmol l^−1^) were used for reaction in 1X SYBR Green Mixture (ABI) in a total volume of 50 μl. The sequences of primers used in the real-time PCR experiment were shown (Supplementary Table 7).

### Immunofluorescence

For immunofluorescence staining, cells on glass coverslips were fixed in 4% formaldehyde, washed with PBS and permeated with 0.01% Triton X-100 (refs 38, 56). After washing with PBS, samples were incubated with blocking solution for 1 h, followed by 1 h of incubation with an anti-E-cadherin antibody or an anti-vimentin antibody. FITC-conjugated goat anti-rabbit immunoglobulin G (IgG) and rhodamine-conjugated goat anti-mouse IgG was used to visualize the location of E-cadherin and vimemtin, respectively. Cell nuclei of cultures were counterstained with Hoechst 33342 (Sigma-Aldrich Corp., St Louis, MO, USA) and fluorescence images were captured using a Leica Laser scanning confocal microscope.

### *In vitro* migration/invasion and tail vein metastatic assay

For *in vitro* migration/invasion assay, 8-μm pore size Boyden chamber was used^38^,^56^. Cells (1 × 10^5^) in 0.5% serum-containing RPMI were plated in the upper chamber and 15% fetal bovine serum was added to RPMI 1640 in the lower chamber as a chemoattractant. For invasion assay, the upper side of the filter was covered with Matrigel (Collaborative Research Inc., Boston, MA, USA) (1:3 dilution with RPMI). After 12 h for migration assay or 24 h for invasion assay, cells on the upper side of the filter were removed, and cells that remained adherent to the underside of membrane were fixed in 4% formaldehyde and stained with Hoechst 33342 dye. The number of migrated cells was counted using a fluorescence microscope. Ten contiguous fields of each sample were examined using a × 40 objective to obtain a representative number of cells which migrated/invaded across the membrane. For *in vivo* tail vein metastasis assay, 6-week old female nonobese diabetic-severe combined immunodeficiency mice received injection of 4 × 10^6^ cells of different cell lines in 0.1 ml of PBS via the tail vein (six mice for each group). Four to six weeks after injection, mice were examined grossly at necropsy for the presence of metastases in internal organs such as lung tissue and lymph node area. Microscopic examination of metastases was performed on the cross-sections of formalin-fixed, paraffin-embedded lung tissues stained with haematoxylin and eosin. The counting of metastatic lesions in the internal organ of each mouse was evaluated by gross and microscopic examination. For orthotopic transplantation assays, 1 × 10^6^ cells of different FADU clones in 0.1 ml of PBS were inoculated into the dorsal side of the left tongue. Mice were killed 6 weeks after implantation. The counting of metastatic lesions in the cervical area and internal organ (lung tissue) of each mouse was evaluated by gross and microscopic examination. Six mice were used for each group of experiment. This study was approved by the Ethics committee of Taiwan Advance Biopharm, Inc (TABP).

### Transient transfection and luciferase assays

The reporter construct was co-transfected into 293T cells with different expression vectors under normoxic/hypoxic culture^38^,^56^. A pCMV-β-gal plasmid was used as an internal control. Luciferase assays were performed using the same amount of cell extracts and corrected for transfection efficiency using internal controls (β-gal).

### Quantitative chromatin immunoprecipitation

Cells were cross-linked with 1% formaldehyde for 10 min and stopped by adding glycine to a final concentration of 0.125 M^56^. Fixed cells were washed twice with Tris buffered saline (20 mM Tris, pH 7.5, 150 mM NaCl) and harvested in 5 ml of SDS buffer (50 mM Tris, pH 8.0, 0.5% SDS, 100 mM NaCL, 5 mM EDTA and protease inhibitors). Cells were pelleted by centrifugation and suspended in 2 ml of IP buffer (100 mM Tris, pH 8.6, 0.3% SDS, 1.7% Triton X-100, 5 mM EDTA). Cells were sonicated with a 0.25-inch diameter probe for 15 s twice using an MSE-soniprep 1500 sonicator (setting 18). For each immunoprecipitation, 1 ml of lysate was precleared by adding 50 μl of blocked protein A beads (50% protein A-Sepharose, Amersham Biosciences; 0.5 mg ml^−1^ bovine serum albumin, 0.2 mg ml^−1^ salmon sperm DNA) at 4 °C for 1 h. Samples were spun, and the supernatants were incubated at 4 °C for 3 h with no antibody, IgG or antibodies to be tested. Immune complexes were recovered by adding 50 μl of blocked protein A beads and incubated overnight at 4 °C. Beads were successively washed with (1) mixed micelle buffer (20 mM Tris, pH 8.1, 150 mM NaCl, 5 mM EDTA, 5% w/v sucrose, 1% Triton X-100, 0.2% SDS), (2) buffer 500 (50 mM Hepes, pH 7.5, 0.1% w/v deoxycholic acid, 1% Triton X-100, 500 mM NaCl, 1 mM EDTA), (3) LiCl detergent wash buffer (10 mM Tris, pH 8,0.5% deoxycholic acid, 0.5% Nonidet P-40, 250 mM LiCl, 1 mM EDTA), and (4) TE buffer (10 mM Tris, 1 mM EDTA) and then eluted with 1% SDS and 0.1 M NaHCO_3_. 20 μl of 5 M NaCl was added to the elutes, and the mixture were incubated at 65 °C for 4 h to reverse the cross-linking. After digestion with proteinase K, the solution was phenol/chloroform-extracted and ethanol-precipitated. DNA fragments were resuspended in 50 μl of water and 5 μl was used in a PCR reaction. For qChIP assay, DNA samples were quantified by the SYBR Green assay using SYBR Green PCR Master Mix (Applied Biosystems) with specific primer. Data were analysed by the CT method and plotted as % input DNA. qChIP values were calculated by the following formula: % input recovery =[100/(input fold dilution/bound fold dilution)] × 2^(input CT-bound CT)^. The antibodies used are listed (Supplementary Table 6). The primers used in qChIP assay are listed (Supplementary Table 8).

### Lentivirus siRNA experiments

Lentivirus containing short hairpin RNAs (shRNAs) expressed in a lentiviral vector (pLKO.1-puro) were generated in 293T cells^57^. For lentivirus production, 293T cells were transfected with 15 μg pLKO.1-puro lentiviral vectors expressing different shRNAs along with 1.5 μg of envelope plasmid pMD.G and 15 μg of packaging plasmid pCMVΔR8.91. Virus was collected 48 h after transfection. The sequence and clonal names of lasmid pLKO-scrambled or other pLKO plasmids against HAUSP, HectH9, TRAF6 and VHL were described in Supplementary Table S8. These LKO plasmids and packaging plasmid pCMVΔR8.91 were provided by National RNAi Core Facility of Academia Sinica (Taipei, Taiwan) or obtained from S.C. Teng (National Taiwan University, Taiwan). To prepare HAUSP, HectH9, TRAF6 or VHL knockdown cells, PC3 or H1299 cells were infected with lentivirus for 24 h, and stable clones were generated by selection with appropriate antibiotics. The sequences used in RNAi experiments were shown (Supplementary Table 9).

### Protein in-gel digestion

The purified HAUSP7 protein was loaded into 10% SDS-PAGE and stained by commassie blue. The gel was cut and each gel slice was cut into small pieces and washed with 25 mM triethylammonium bicarbonate (TEABC) in 50% (v/v) ACN for 15 min. The wash step was repeated three times. Gel slices were dehydrated with 100% acetonitrile (ACN) and vacuum-dried for 10 min. The dried gel pieces were rehydrated with 25 mM TEABC, reduced the disulfide bonds with 5 mM TCEP-HCl for 30 min at 37 °C and alkylated the free thiol groups with 10 mM iodoacetamide (IAM) for 1 h vortexing at room temperature. After removing the reagents, the gel pieces were washed three times with 25 mM TEABC in 50% (v/v) ACN for 15 min and dehydrated with 100% ACN and vacuum-dried for 10 min. The dried gel pieces were rehydrated with 25 mM TEABC and trypsin/chymotrypsin (1:25 w/w, enzymes: proteins) was added. The mixture was incubated at 37 °C for 16 h. The digested peptides were extracted twice with 5% (v/v) FA in 50% (v/v) ACN for 30 min and dried completely under vacuum. The peptides were resuspended in 0.1% TFA and desalted by C_18_ Zip-tip (Millipore) and subjected to downstream MS analysis.

### LC-ESI-MS/MS analysis

NanoLC-MS/MS analysis was performed on a Thermo Scientific UltiMate 3000 RSLCnano system configured with a CTC PAL autosampler connected to an Thermo Orbitrap Fusion mass spectrometer (Thermo Fisher Scientific, Bremen, Germany) equipped with a nanospray interface. Peptide mixtures were directly loaded onto a 30 cm self-pulled column with a 100 μm inner diameters and 7 μm opening in-house reversed phase analytical column that were prepared 3 μm ReproSil-Pur C18-AQ particles (Dr Maisch, Ammerbuch, Germany). Mobile phase buffer A was composed of water with 0.1% formic acid and a segmented gradient in 100 min from 2 to 85% buffer B (acetonitrile with 0.1% formic acid) at 500 nl min^−1^ flow rate. The mass spectrometer was operated in the data-dependant mode. Briefly, survey scans of peptide precursors from 300 to 1600 m/z were performed at 120 K resolution with a 5 × 105 ion count target. Tandem MS was performed by isolation window at 1.6 Da with the quadrupole, CID fragmentation with normalized collision energy of 30, and rapid scan MS analysis in the ion trap. The MS2 ion count target was set to 104 and the max injection time was 50 ms. Only those precursors with charge state 2–6 were sampled for MS2. The instrument was run in top speed mode with 3 s cycles; the dynamic exclusion duration was set to 60 s with a 10 ppm tolerance around the selected precursor and its isotopes. Monoisotopic precursor selection was turned on.

### Protein identification

The peak list resulting from the MS/MS spectra was exported to mgf format using Mascot Distiller v2.1.1.0 by the following criteria: the precursor peak charge range on MS and MS/MS processing was 2 to 5; the precursor mass range was 400–4000 and the precursor m/z tolerance was 0.3; S/N≥3. The data sets were batch-searched and combined searched with Mascot v2.2 (Matrix Science, London, UK) against the SwissProt database (v57.8, total 509,019 sequences, 20,329 of which are Homo sapiens) using the following constraints: only tryptic peptides with up to two missed cleavage sites were allowed, and the mass tolerance for both peptides and MS/MS fragment ions was 0.1 Da. DiGly modification on Lys (+114.04 Da), carbamidomethyl on Cys, and oxidation on Met were specified as variable modifications. Peptides were considered to be identified if their Mascot individual ion score was higher than the Mascot identity score (*P*<0.05). To evaluate the false discovery rate in protein identification, a decoy database search against a randomized decoy database created by Mascot using identical search parameters, and validation criteria was also performed.

### Nuclear/cytoplasmic fractionation

The subcellular fractionation of cells was performed using a ProteoJET cytoplasmic and nuclear protein extraction kit (Fermentas). Cells were collected by using a cell scraper, lysed with Cell lysis buffer containing protease inhibitors and then incubated on ice for 10 min. Permeabilized cells were subsequently centrifuged at 500*g* for 7 min at 4 °C. The pellet was put on ice and the supernatant was further centrifuged at 20,000*g* for 15 min at 4 °C. The 20,000*g* supernatant was collected and referred to as the cytoplasmic fraction. After washing the pellet with Nuclei washing buffer, Nuclei lysis buffer was added to the pellet and the sample was incubated on ice for 15 min. After centrifugation at 20,000 × *g* for 5 min at 4 °C, the supernatant was collected and referred to as the nuclear fraction.

### Co-immunoprecipitation and GST pull down assays

Co-immunoprecipitation experiments were performed by incubating different antibodies (Supplementary Table 6) for 5 h with 0.5–1 mg of whole cell lysates prepared by lysis in 150 mM NaCl, 1% Nonidet P-40, 1% deoxycholate, 0.1% SDS, 50 mM Tris HCl (pH 7.5), 1 mM PMSF, 25 mM NaF and protease inhibitors, from 293T cells overexpressing proteins of interest^38^,^56^. The immune complexes were incubated overnight with protein-A beads, preblocked with 10% bovine serum albumin. The immunoprecipitates were washed three times with the same lysis buffer, mixed with 1 × Laemmli dye, boiled for 5 min, and loaded on SDS-PAGE. After transfer, the filters were blocked with blocking buffer, probed with primary and secondary antibody sequentially and developed. GST pull down assays were performed by incubating His-HIF1α1–400 or His-HIF1α 401–603 protein with GST-HAUSP1–209 or GST-HAUSP210–500 protein (or GST protein as a control) and Glutathione-Agarose (Sigma, St Louis, MI, USA). Purification of GST proteins was described. The pulled down His-HIF1α protein was detected by Western blot analysis.

### Deubiquitination assays

For *in vivo* deubiquitination assay, 293T cells were transfected with the indicated plasmids. After 24 h, the cells were incubated under normoxic or hypoxic condition with a proteasome inhibitor, MG132 (Sigma) (50 mM) for 16 h and lysed by RIPA buffer. The cell extracts were incubated with nickel-beads for 6 h, washed by RIPA buffer and subjected to Western blot analysis. For *in vitro* deubiquitination assay, Flag-HAUSP wild type and its mutants were expressed in 293T cells under normoxic or hypoxic condition, immunoprecipitated by anti-Flag agarose (Sigma), and eluted by Flag peptides (100 mg ml^−1^) in the deubiquitination buffer (50 mM Tris-HCl pH 8.0, 50 mM NaCl, 1 mM EDTA, 10 mM DTT, 5% glycerol). The (His)6-ubiquitinated HA-HIF-1a were expressed in 293T cells with MG132 treatment, precipitated by Nickel beads (Sigma) and eluted by glutathione (100 mM) in the deubiquitination buffer. Purified (His)6-ubiquitinated HA-HIF-1α, Flag-HAUSP wild type and its mutants were incubated in a deubiquitination buffer for 15–30 min at 37 °C and subjected to Western blot analysis.

### Histone acetyltransferase assays

293T cells were transiently transfected with the plasmids of Flag-CBP, HA-HIF-1α and Flag-HAUSP. Following separation of histone extracts, western blotting was performed to detect H3 and H3K56Ac. For *in vitro* HAT assay, the HAUSP WT, K443R mutant and polyubiquitinated HAUSP WT proteins were purified by anti-Flag conjugated agarose from 293T cells transiently transfected with HAUSP WT/Mut and ubiquitin plasmids. The Flag-CBP WT, ΔZZ-TAB2 and ΔZZ were also purified by anti-Flag conjugated agarose. The commercial human recombinant Histone H3 proteins (Caymen, No. 10263) were used in the experiment. Acetylation reactions were performed for 0.5–1 h at 30 °C in 50 mM Tris-HCl, pH 8.0, 10% glycerol, 1 mM DTT, 1 mM PMSF and 0.1 mM EDTA. Western blot analysis was performed to detect H3, H3K56Ac and H3K18Ac.

### Protein complex modelling

The protein complex comprised H3, CBP and HAUSP with polyubiquitin. The template of HAUSP is based on the chain A structure of PDB ID 2F1Z from residue no. 64 to 553 (ref. 58). The template of CBP is composed of several structures including the chain A of PDB ID 4BHW from residue no. 1082 to no. 1702 (ref. 59), the chain A of PDB ID 4XI6 from residue no. 1704 to no. 1745 and the chain A of PDB ID 3T92 from residue no. 1761 to no. 1867 (ref. 60). The polyubiquitin is modelled based on the chain A structure of PDB ID 1UBQ from residue no. 1 to no. 76 (ref. 61). The template of H3 is based on the chain A structure of PDB ID 3AV1 from residue no. 39 to no. 134 (ref. 62). The popular molecular graphics system PyMOL was used to build the protein complex model. The initiated related positions of all structures were placed by user-defined parameters. The optimization of the delicate related positions was manually based on the protein surface charge, which were calculated by PyMOL.

### ChIP-seq data analysis

The DNA obtained from the HAUSP- and HIF-1α-ChIP assays were sequenced by Illumina Hiseq 2000. The public HIF-1α ChIP-seq data set was obtained from Gene Expression Omnibus (GSE39089)^63^. Raw sequencing reads were mapped to the human genome (assembly hg19) using Bowtie v0.12.7 algorithm with m1 and v3 parameters^64^. The binding regions of HAUSP and HIF-1α were identified by MACS v1.4.2 algorithm^65^ with default parameter and then annotated by the Bioconductor package, ChIPpeakAnno^66^.

### Gene ontology analysis of ChIP-seq data

The genes with peaks located around the TSS (from −500 to 500 bp) were chosen for further analysis. Genes were chosen through the criterion from each ChIP-seq data set (532 genes from HAUSP, 107 from our HIF1 and 837 from the public HIF-1α ChIP-seq). There are 31 genes identified in both HAUSP and our HIF-1α ChIP-seq, and 79 genes found in both HAUSP and the public HIF-1α ChIP-seq data sets. We used GO-based functional enrichment analysis to measure the gene-enrichment in annotation terms for selected genes by Fisher's Exact test. The genes in the GO terms that passed the criteria of *P* value <0.01 and at least two genes in each GO term were considered for further analysis.

### HAUSP motif finding and scanning

The DNA sequences of HAUSP peaks in the 31 overlapped genes between HAUSP and our HIF-1α ChIP-seq along with 500 bp extensions from both ends were used for motif finding. Two motifs, motif-1 and motif-2, were identified by the motif discovery analysis performed by MEME-ChIP^67^. The DNA sequences from −5000 to 500 bp from the TSS of the interested genes were used for identification of the putative motif binding site of the motif-1 and motif-2 by FIMO^68^.

### *In vitro* ubiquitination assay

Flag-HAUSP wild type and K443R mutants were overexpressed respectively in 293T cells and immunoprecipitated by anti-Flag agarose (Sigma). The v5-HectH9 was overexpressed in 293T cells, immunoprecipitated by anti-v5-antibodies (Invitroen) and eluted by v5 tag peptide (10 μg ml^−1^) in the PBS. The commercial ubiquitination kit (Enzo Life Science, BML-UW9920) was used to perform this experiment. The reaction was incubated for 4-8 h at 37 °C and subjected to Western blot analysis by using anti-K63 ubiquitin antibodies.

### Study population and sample collection

One hundred and ninety stage I lung cancer patients who underwent surgical resection at Taipei Veterans General Hospital between January 2002 and December 2006 were retrospectively analysed. Informed written consent was obtained from all the patients. This study has been approved by the Institutional Review Board of Taipei Veterans General Hospital. The median follow-up duration was 54.6 months (range 4.7∼100 months). The clinical characteristics of 190 lung adenocarcinoma patients are illustrated (Supplementary Table 2). Primary tumour samples and the corresponding non-cancerous matched tissue were obtained during surgery. A high-density tissue microarray was constructed using early stage non-small cell lung adenocarcinoma patient samples^38^,^56^. Two pathologists evaluated tissue sections by routine haematoxylin and eosin staining and identified the regions of interest for IHC analysis.

### Immunohistochemistry and scoring

Six μm thick sections of tumour tissue were cut from the frozen specimens for IHC analysis^38^,^56^. The samples were fixed in acetone, air-dried and subsequently bathed in Tris buffered saline solution (pH 7.6). The endogenous peroxidase activity was blocked with 3% hydrogen peroxide. After reacting with a biotinylated secondary antibody for 30 min, antigen-antibody reactions were visualized using streptavidin-horseradish peroxidase conjugate (DAKO LSAB kit; DAKO, Los Angeles, CA, USA), with 3-amino-9-ethylcarbazole as the chromogen. All slides were counterstained with haematoxylin. All IHC staining was independently scored by two experienced pathologists. If there was discordance with IHC scoring, a pathologic peer review will be performed to consolidate the result into a final score. The pathologists scoring the IHC were blinded to the clinical data. The interpretation was performed in five high power views for each slide, and 100 cells per view were counted for analysis. The antibodies used in IHC are listed (Supplementary Table 6).

### Statistical analyses

Unless otherwise noted, each sample was assayed in triplicate. For *in vitro* analyses, each experiment was repeated at least three times. All error bars represent s.d. Student's *t* test was used to compare two groups of independent samples. For clinical samples, the *X*^2^ test was applied for comparison of dichotomous variables. The Kaplan-Meier estimate was used for survival analysis, and the log-rank test was used to compare the difference. The level of statistical significance was set at 0.05 for all tests.

### Data availability

The Gene Expression Omnibus accession number for the ChIP-seq data reported in this paper is GSE76770. All the remaining data, not included in the manuscript or Supplementary Information, that support the findings of this study are available from the corresponding author on request.

## Additional information

**How to cite this article:** Wu, H.-T. *et al*. K63-polyubiquitinated HAUSP deubiquitinates HIF-1α and dictates H3K56 acetylation promoting hypoxia-induced tumour progression. *Nat. Commun.* **7**, 13644 doi: 10.1038/ncomms13644 (2016).

**Publisher's note:** Springer Nature remains neutral with regard to jurisdictional claims in published maps and institutional affiliations.

## Supplementary Material

Supplementary Information Supplementary Figures 1-7, Supplementary Tables 1-9, Supplementary Notes.

Supplementary Data 1 The file is the excel file that contains the information of ChIP-seq analysis and the genes containing motif-1 and motif-2 in their promoters.
