# Supplementary material for: K63-polyubiquitinated HAUSP deubiquitinates HIF-1α and dictates H3K56 acetylation promoting hypoxia-induced tumour progression
Source: Nat Commun. 2016 Dec 9;7:13644. doi: 10.1038/ncomms13644 (PMC5155157; doi:10.1038/ncomms13644)

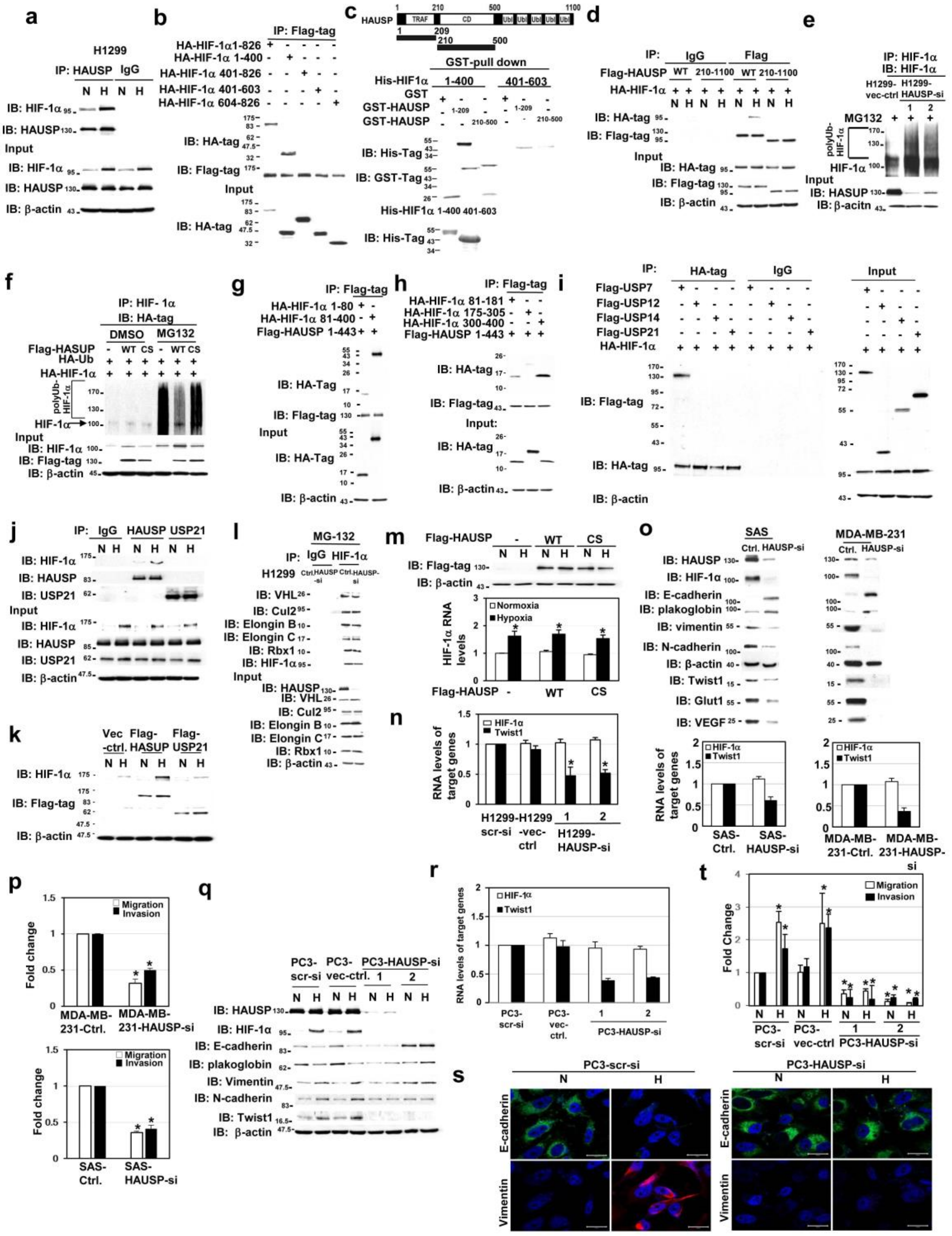

Supplementary Figure 1. Demonstration of interaction between HAUSP and HIF-1α and various functional characterizations of HAUSP. (a)-(d) Demonstration of interaction between HAUSP and HIF-1α and mapping of their interacting domains by co-immunoprecipitation assays. **(e) & (f)** HAUSP levels

correlated with the polyubiquitinated HIF-1α levels in H1299 cells in knockdown or overexpression experiments under MG132 treatment. MG132 was used to inhibit proteasome-mediated HIF-1α degradation. **(g)-(h)** Further mapping of the domain in HIF-1α that interacted with the a.a. 1-443 domain of HAUSP by co-expressing experiments in 293T cells using co-immunoprecipitation assays. **(i)-(k)** Characterizations of other USP family members that did not interact with or stabilize HIF-1α. **(l)** Co-immunoprecipitation assays showed that knockdown of HAUSP in H1299 cells did not affect the interaction between HIF-1α and the VHL/E3 ubiquitin ligase complex in H1299 cells. **(m) & (n)** Overexpression or knockdown of HAUSP did not affect the mRNA levels of HIF-1α under hypoxia by real time PCR analysis**.** No HAUSP-transfected (normoxia) or H1299 scrambled-siRNA sample was used as the control for statistical analysis. **(o) & (p)** Knockdown of HAUSP reversed EMT, decreased HIF-1α target gene expression with no alteration in HIF-1α mRNA levels, and decreased the *in vitro* migration and invasion activity in two cell lines. The scrambled-siRNA controls were used as the controls for statistical analysis for either SAS or MDA-MD-231 cells. **(q)** Western blot analysis of epithelial and mesenchymal markers under normoxia and hypoxia in HAUSP-knockdown PC3 and control PC3 cells (PC3 scrambled-siRNA control cells and PC3 vector control cells). **(r)** Knockdown of HAUSP reduced the *Twist1* mRNA levels but did not affect the endogenous *HIF-1α* mRNA levels in PC3 cells. The PC3 scrambled-siRNA controls were used as the controls. Green fluorescence: E-cadherin; red fluorescence: vimentin. **(s)** & **(t)** Knockdown of HAUSP reversed the EMT marker changes by immunofluorescence assay or decreased the *in vitro* migration and invasion activity induced by hypoxia in PC3 cells. The PC3 scrambled-siRNA control clone was used as the control. Anti-IgG antibodies were used as negative controls in co-immunoprecipitation assays. For **(m)-(p), (r) & (t)**, data from three independent experiments are expressed as mean ± s.d. The asterisk (*) indicates statistical significance ($P<0.05$) between experimental and control clones/transfections, t-test. N: normoxia, H: hypoxia.

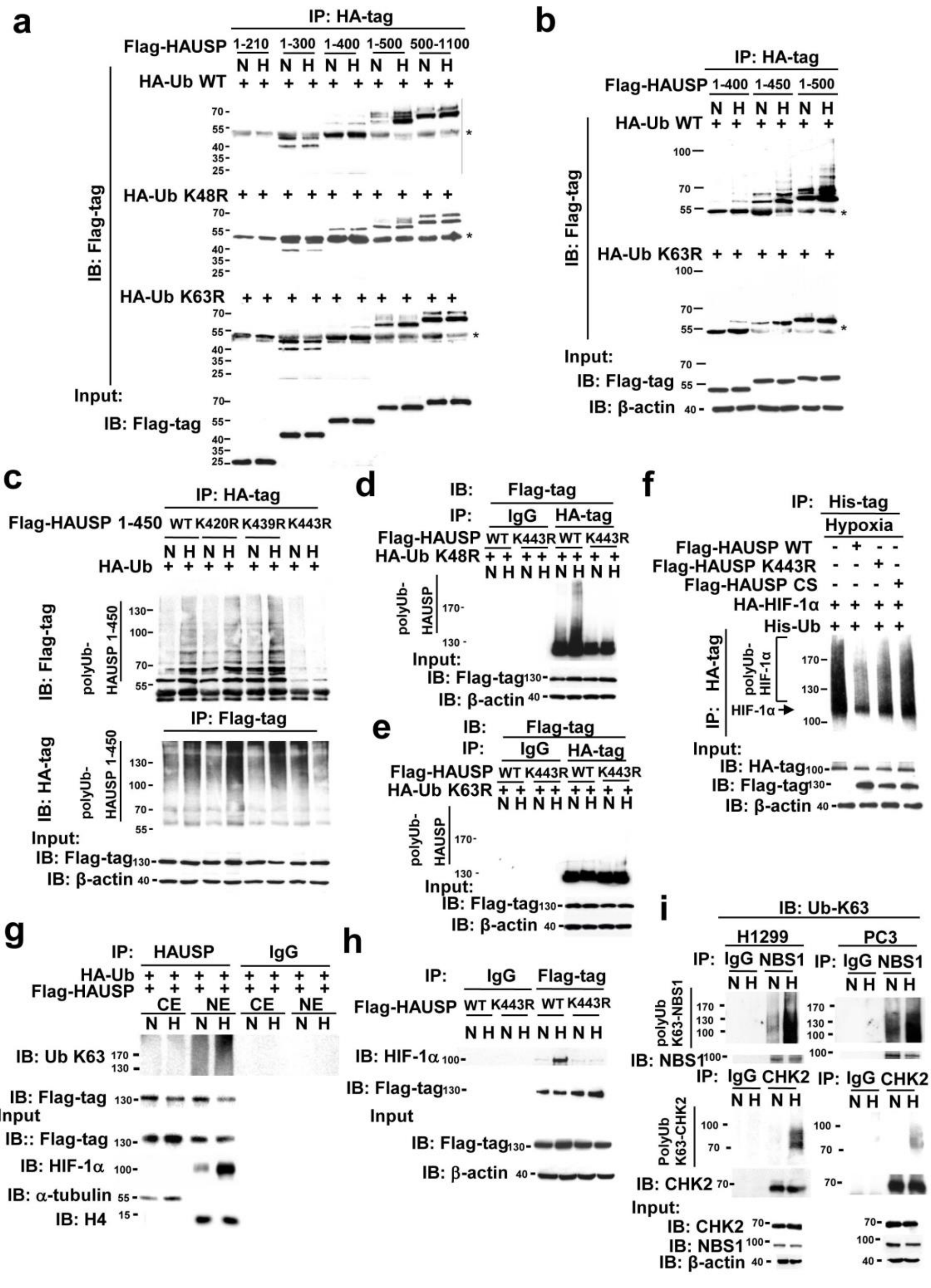

Supplementary Figure 2. Increase in the deubiquitinase activity of HAUSP by

hypoxia and mapping of the K63-polyubiquitinated site of HAUSP. (a) & (b) Domain mapping experiments showed that the a.a. 400-450 domain in HAUSP is K63-linked polyubiquitinated under hypoxia, as demonstrated by expressing different truncation mutants of HAUSP and HA-Ub in 293T cells followed by the pull-down with anti-HA antibodies. The asterisk (*) indicates heavy chain. **(c)** K443 was the site responsible for hypoxia-induced K63-linked polyubiquitination. Co-expression of HA-ubiquitin and Flag-tagged HAUSP1-450 (wild type, K420R, K439R, or K443R) mutants followed by co-immunoprecipitation experiments showed that the polyubiquitination levels of HAUSP1-450^{K443R} mutant was significantly less than the wild type or other mutants. **(d)** Western blot assays showed that the K48R ubiquitin was conjugated to the full-length wild type HAUSP, but not the HAUSP^{K443R} mutant, by co-immunoprecipitation experiments in 293T cells. **(e)** Western blot assays showed that the K63R ubiquitin was not able to be conjugated into either wild type or mutant HAUSP by co-immunoprecipitation experiments in 293T cells. **(f)** The HAUSP^{K443R} mutant had a significantly less ability to deubiquitinate polyubiquitinated HIF-1α compared to the wild-type HAUSP under hypoxia, as demonstrated by expressing HA-HIF-1α and Flag-HAUSP vector (wild type, K443R, or CS mutant) in 293T cells followed by Western blot analysis. **(g)** Hypoxia-induced K63-linked polyubiquitination of HAUSP was located in the nucleus and further increased under hypoxia as demonstrated by expressing Flag-HAUSP and HA-ubiquitin in 293T cells followed by anti-HAUSP antibodies pull down using Western blot analysis. The cytoplasmic and nuclear fractions were isolated according to the methods described in the method section. **(h)** Co-immunoprecipitation assays using the HAUSP wild type vs. the HAUSP^{K443R} mutant to interact with HIF-1α under hypoxia were shown. Flag-HAUSP (wild type or mutant) was expressed in 293T cells followed by co-immunoprecipitation of endogenous HIF-1α with anti-Flag antibodies. N: normoxia, H: hypoxia. **(i)** Increased K63-linked polyubiquitination status of CHK2 and NBS1 under hypoxia in two different cell lines. Anti-CHK2 or NBS1 antibodies were used to pull down CHK2 or NBS1 under normoxia or hypoxia followed by blotting with antibodies against K63 polyubiquitin chain. Anti-IgG antibodies were used as negative controls in co-immunoprecipitation assays.

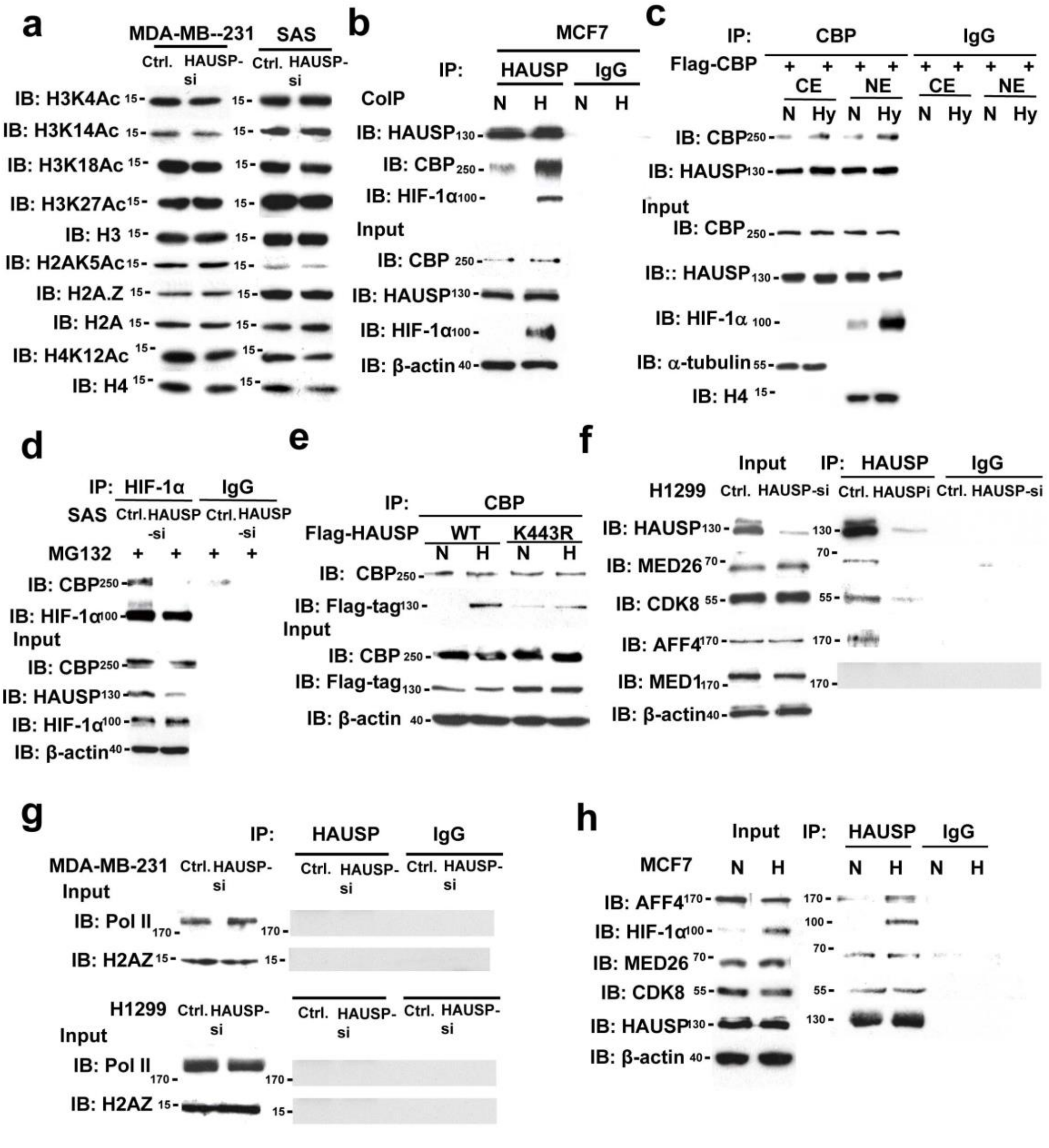

Supplementary Figure 3. Screening of various histone marks in control vs. HAUSP knockdown cell lines and co-immunoprecipitation experiments. (a) Screening of various histone marks in control vs. HAUSP knockdown cell lines (SAS,

MDA-MB-231) by Western blot analysis. **(b)** Co-immunoprecipitation assays showed the increased the interaction between HAUSP and CBP under hypoxia in MCF7 cells. N: normoxia, H: hypoxia. **(c)** Co-immunoprecipitation assays showed that the increased interaction between HAUSP and CBP under hypoxia occurred in the nuclear fraction as demonstrated by expressing Flag-CBP in 293T cells followed by the pull-down with anti-CBP antibodies**.** CE: cytoplasmic extract, NE: nuclear extract. **(d)** Knockdown of HAUSP in SAS cells decreased the interaction between HIF-1α and CBP by co-immunoprecipitation assays. **(e)** Decreased interaction between $HAUSP^{K443R}$ mutant and CBP (compared to wild-type HAUSP) under hypoxia was detected by expressing Flag-HAUSP (wild type or K443R mutant) in 293T cells followed by the pull-down with anti-CBP antibodies using co-immunoprecipitation assays. **(f)** Co-immunoprecipitation assays showed the interaction between HAUSP and CDK8, MED26, AFF4, but not between HAUSP and MED1 in H1299 cells. Knockdown of HAUSP abolished all the interactions. **(g)** No interaction between HAUSP and the Pol II subunit or H2A.Z in two cell lines (MDA-MB-231, H1299) by co-immunoprecipitation assays. **(h)** Increased interaction between HAUSP and AFF4 under hypoxia by co-immunoprecipitation assays. No difference in the interaction between HAUSP and MED26 or CDK8 under normoxia vs. hypoxia was observed. N: normoxia, H: hypoxia. Anti-IgG antibodies were used as negative controls in co-immunoprecipitation assays.

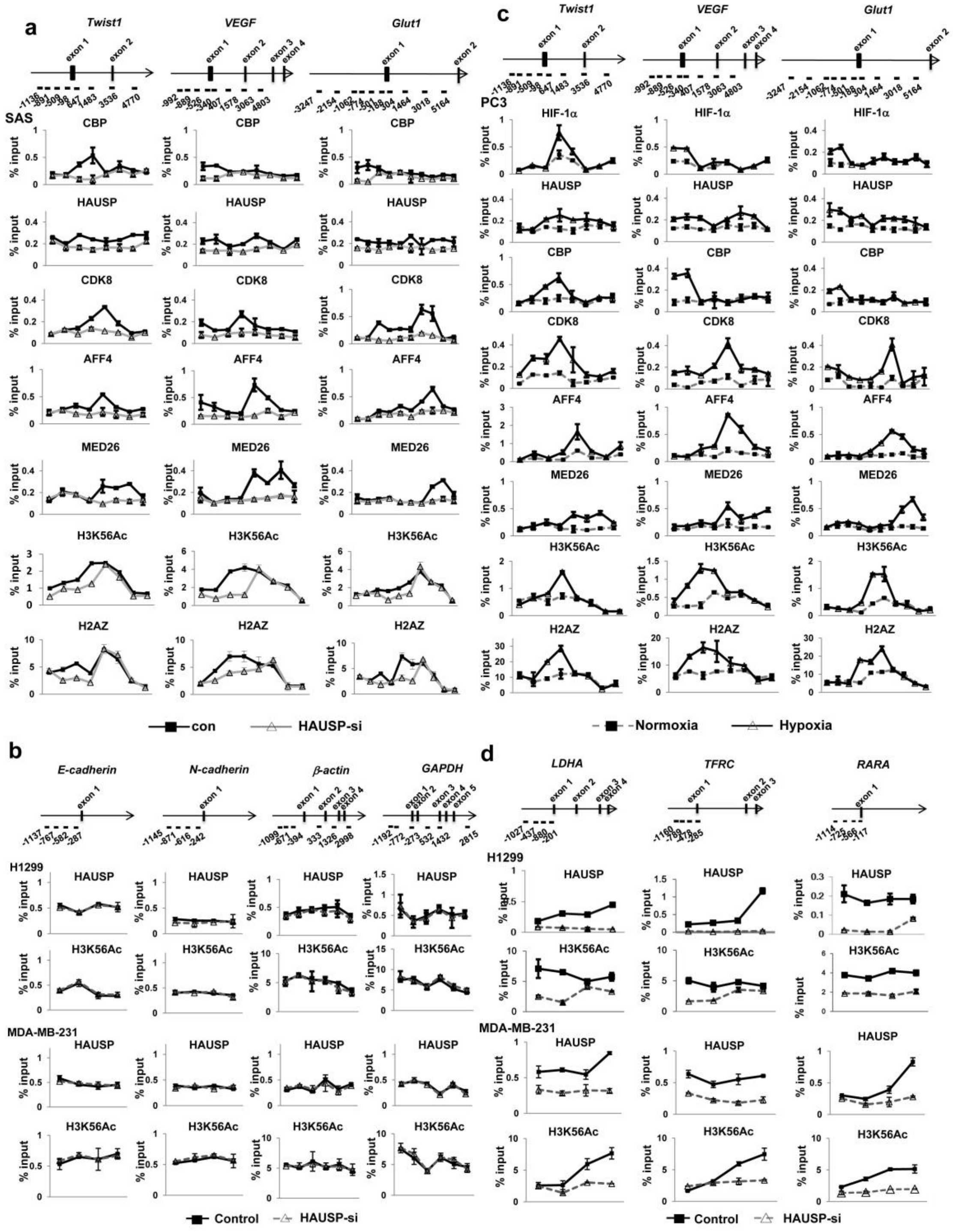

Supplementary Figure 4. qChIP assays of the binding of various proteins as well as the levels of H3K56Ac and H2A.Z on the promoters of various genes. (a)

qChIP levels of the binding of various proteins and histone marks on the promoters/gene body of three genes (*Twist1, VEGF, Glut1*) between control and HAUSP knockdown clone in SAS cells. Different regions located in the promoters and inside the gene body of three genes were shown underneath the plotting of genes. The different numbers indicated the starting nucleotide positions in the regions (labeled by brackets) before and after the initiation site. **(b)** No difference of the qChIP levels of H3K56Ac in the various promoter regions of *E-cadherin*, *N-cadherin*, *β-actin*, and *GAPDH* genes in two different cell lines (H1299, MDA-MB-231). **(c)** qChIP levels of the binding of various proteins and histone marks on the promoters/gene body of three genes (*Twist1, VEGF, Glut1*) between normoxia and hypoxia in PC3 cells. Different regions located in the promoters and inside the gene body of three genes were shown underneath the plotting of genes. The different numbers indicated the starting nucleotide positions in the regions (labeled by brackets) before and after the initiation site. **(d)** qChIP levels of HAUSP binding and H3K56Ac levels on the promoter of three HIF-1α target genes (*LDHA, TFRC, RARA*) between control and HAUSP knockdown clone in two cell lines (H1299, MDA-MB-231). For the qChIP analysis in **(a)**, **(b), (c), & (d)**, data from three independent experiments are expressed as mean ± s.d., *t*-test.

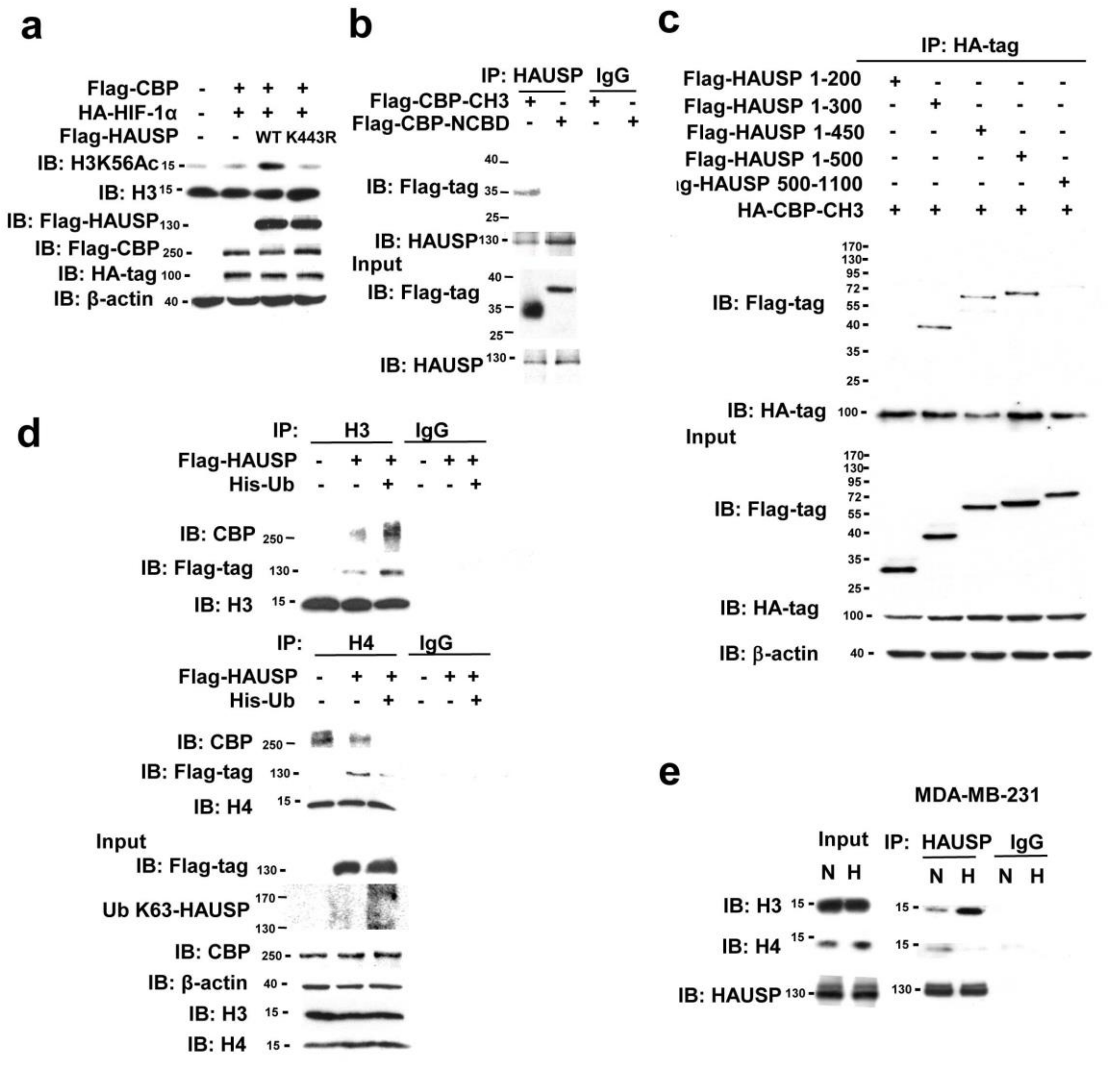

Supplementary Figure 5. Demonstration of the histone acetylase activity of

HAUSP and mapping of its interaction domains with CBP or H3-binding. (a) Overexpression of wild type HAUSP, but not the $HAUSP^{K443R}$ mutant, together with HIF-1α and CBP in 293T cells increased the H3K56Ac levels by Western blot analysis. **(b)** Co-immunoprecipitation assays showed the interaction between HAUSP and the CBP CH3 domain, but not the NCBD domain when two different domains of CBP were respectively expressed in 293T cells followed by the pull-down with anti-HAUSP antibodies. **(c)** Domain mapping experiments showed that a.a. 200-300 region of HAUSP interacted with the CBP CH3 domain by expressing different truncation mutants of HAUSP and the HA-CBP CH3 domain in 293T cells followed by the pull-down with anti-HA antibodies in Western blot analysis. **(d)** Co-immunoprecipitation assays showed the increased binding of the CBP-polyubiquitinated HAUSP complex to histone 3 under hypoxia, but decreased binding of the CBP-polyubiquitinated HAUSP complex to histone 4 under hypoxia. Flag-HAUSP and His-Ub vectors were expressed in 293T cells followed by pull down using anti-H3 or anti-H4 antibodies. **(e)** The interaction between H3/H4 and HAUSP during hypoxia

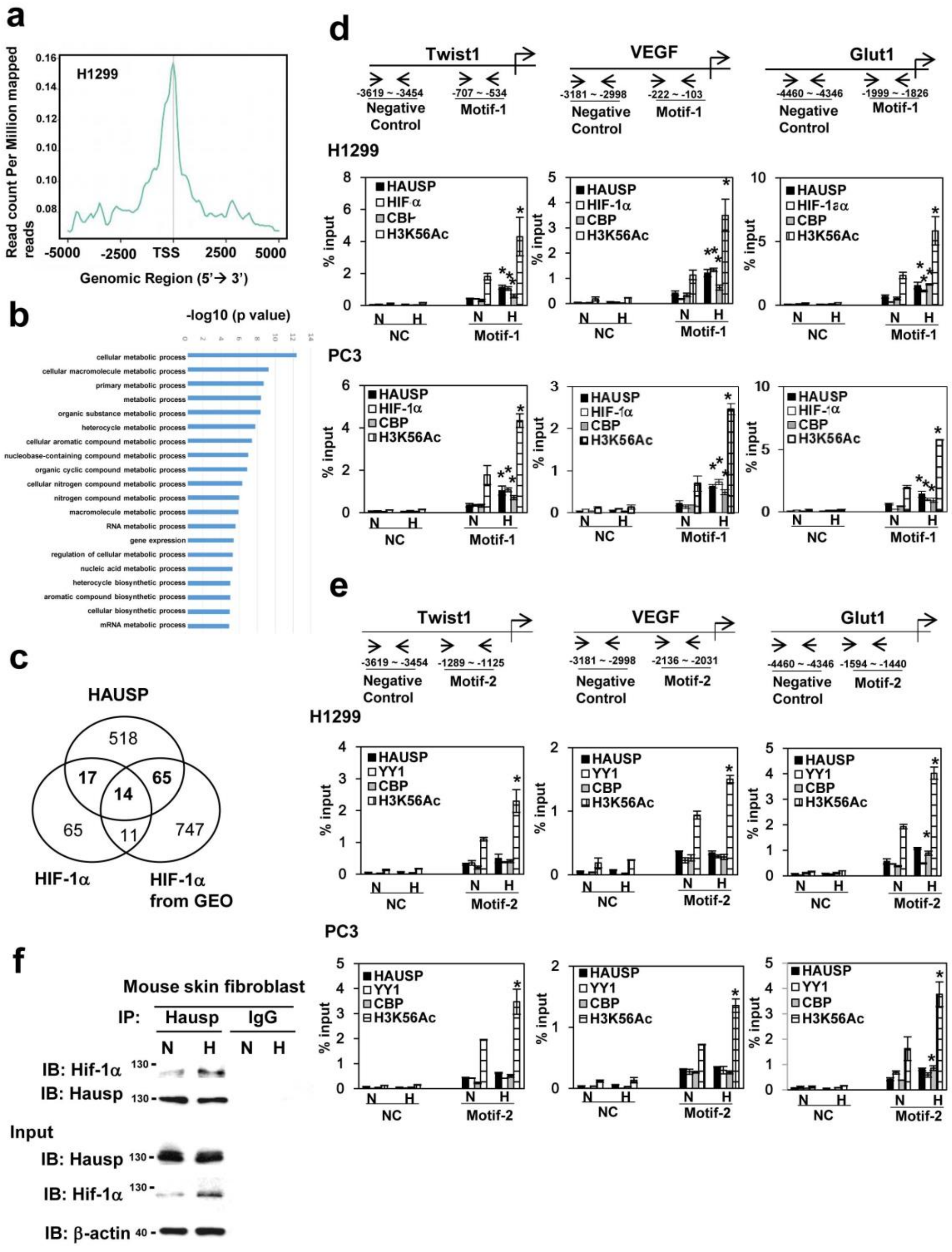
a
H1299
Read count Per Million mapped reads
Genomic Region (5'→ 3')
-5000
-2500
TSS
2500
5000
b
-log10 (p value)
cellular metabolic process
cellular macromolecule metabolic process
primary metabolic process
metabolic process
organic substance metabolic process
heterocycle metabolic process
cellular aromatic compound metabolic process
nucleobase-containing compound metabolic process
organic cyclic compound metabolic process
cellular nitrogen compound metabolic process
nitrogen compound metabolic process
macromolecule metabolic process
RNA metabolic process
gene expression
regulation of cellular metabolic process
nucleic acid metabolic process
heterocycle biosynthetic process
aromatic compound biosynthetic process
cellular biosynthetic process
mRNA metabolic process
c
HAUSP
518
17
14
65
65
11
747
HIF-1α
HIF-1α from GEO
d
Twist1
-3619 ~ -3454
Negative Control
-707 ~ -534
Motif-1
VEGF
-3181 ~ -2998
-222 ~ -103
Glut1
-4460 ~ -4346
-1999 ~ -1826
H1299
PC3
HAUSP
HIF-1α
CBP
H3K56Ac
% input
N
H
NC
Motif-1
e
-1289 ~ -1125
Motif-2
-2136 ~ -2031
-1594 ~ -1440
YY1
f
Mouse skin fibroblast
IP:
Hausp
IgG
IB: Hif-1α
IB: Hausp
Input
IB: β-actin
130
40

Supplementary Figure 6. ChIP-seq results of HAUSP and HIF-1α and qChIP analysis of various factors binding to motifs-1 and 2 in various gene promoters. **(a)** The distrubtion of HAUSP binding around the TSS site using ChIP-seq analysis. **(b)** GO analysis of the 532 genes that showed binding by HAUSP. **(c)** Overlapping set of genes bound by HAUSP and HIF-1α (the set performed in this report as well as the public HIF-1α ChIP-seq data set). **(d) & (e)** qChIP results of the *Twist1, VEGF*, and *Glut1* gene promoters under similar analysis (as in Fig. 6) of motif-1 and motif-2 sites bound by HAUSP, HIF-1α, CBP, and H3K56Ac in H1299 and PC3 cells. The binding of various factors to motif-1 or motif-2 sites under normoxia was used as controls. For **(d) & (e),** data from three independent experiments are expressed as mean ± s.d. The asterisk (*) indicated statistical significance (P<0.05) between hypoxia and normoxia. *t*-test. **(f)** Co-immunoprecipitation assay showed that HIF-1α was expressed in mouse skin fibroblast under normoxia and interacted with HAUSP. Anti-IgG antibodies were used as negative controls in co-immunoprecipitation assays.

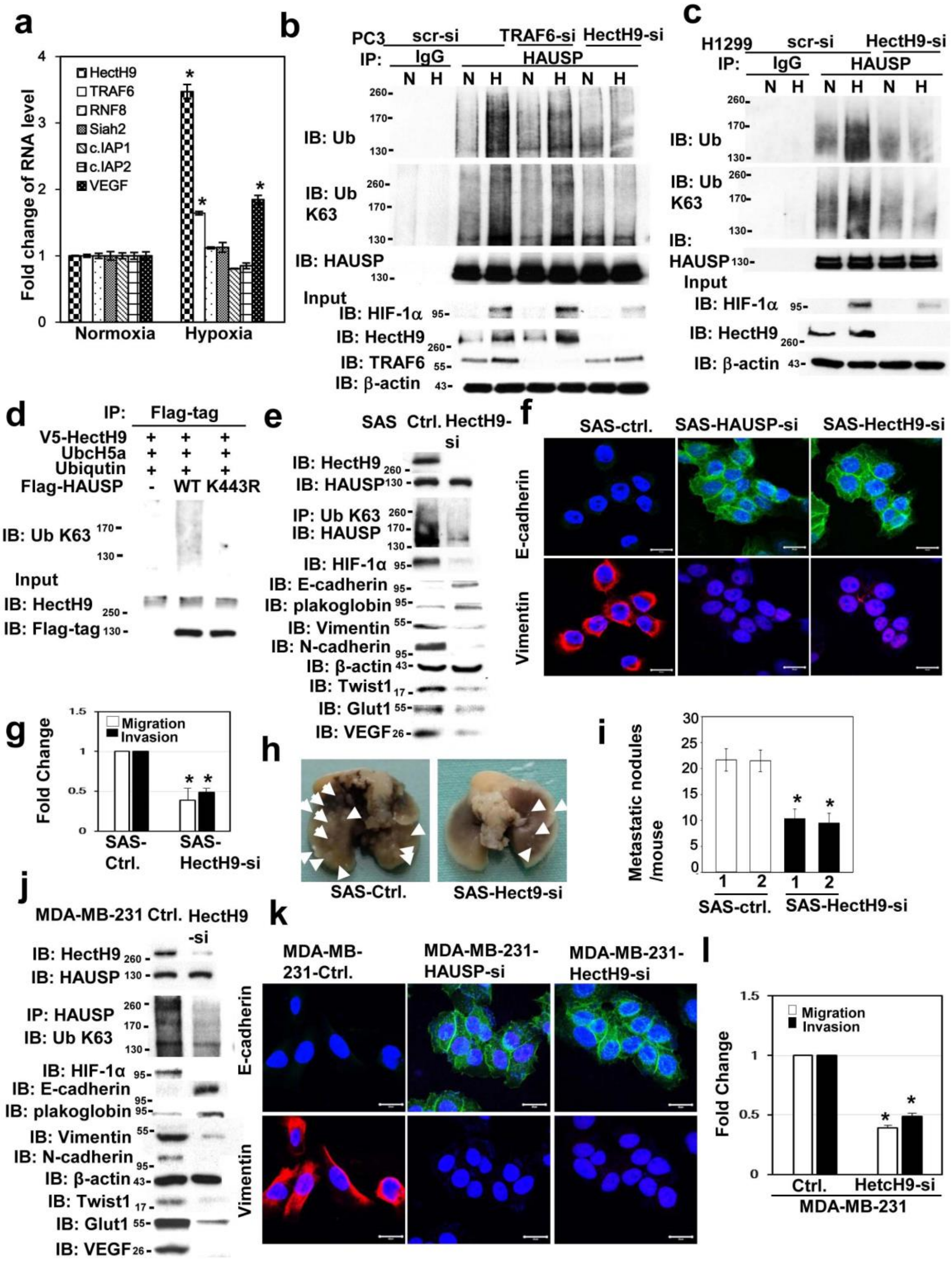
a
Fold change of RNA level
HectH9
TRAF6
RNF8
Siah2
c.IAP1
c.IAP2
VEGF
Normoxia
Hypoxia
b
PC3 scr-si TRAF6-si HectH9-si
IP: IgG HAUSP
N H N H N H N H
IB: Ub
IB: Ub K63
IB: HAUSP
Input
IB: HIF-1α
IB: HectH9
IB: TRAF6
IB: β-actin
c
H1299 scr-si HectH9-si
IP: IgG HAUSP
IB: Ub
IB: Ub K63
IB: HAUSP
Input
IB: HIF-1α
IB: HectH9
IB: β-actin
d
IP: Flag-tag
V5-HectH9
UbcH5a
Ubiqutin
Flag-HAUSP - WT K443R
IB: Ub K63
Input
IB: HectH9
IB: Flag-tag
e
SAS Ctrl. HectH9-si
IB: HectH9
IB: HAUSP
IP: Ub K63
IB: HAUSP
IB: HIF-1α
IB: E-cadherin
IB: plakoglobin
IB: Vimentin
IB: N-cadherin
IB: β-actin
IB: Twist1
IB: Glut1
IB: VEGF
f
SAS-ctrl. SAS-HAUSP-si SAS-HectH9-si
E-cadherin
Vimentin
g
Fold Change
Migration
Invasion
SAS-Ctrl.
SAS-HectH9-si
h
SAS-Ctrl.
SAS-Hect9-si
i
Metastatic nodules /mouse
1 2 1 2
SAS-ctrl.
SAS-HectH9-si
j
MDA-MB-231 Ctrl. HectH9 -si
IB: HectH9
IB: HAUSP
IP: HAUSP
IB: Ub K63
IB: HIF-1α
IB: E-cadherin
IB: plakoglobin
IB: Vimentin
IB: N-cadherin
IB: β-actin
IB: Twist1
IB: Glut1
IB: VEGF
k
MDA-MB-231-Ctrl.
MDA-MB-231-HAUSP-si
MDA-MB-231-HectH9-si
E-cadherin
Vimentin
l
Fold Change
Migration
Invasion
Ctrl.
HetcH9-si
MDA-MB-231

Supplementary Figure 7. The role of HectH9 in mediating K63-linked HAUSP polyubiquitination and inducing EMT and *in vivo* metastatic activity. (a) Expression of HectH9 and TRAF6 was induced by hypoxia using real-time PCR analysis. The mRNA levels of each gene under normoxia were used as the controls for statistical analysis. **(b)** Western blot analysis showed the decreased K63-linked polyubiquitination levels of HAUSP in PC3 cells under HectH9 knockdown when extracts were immunoprecipitated by anti-HAUSP antibodies followed by blotting with anti-Ub or anti-K63-Ub antibodies. scr-si: scrambled siRNA control. Knockdown of HectH9, but not TRAF6, significantly decreased the induction of HIF-1α by hypoxia in PC3 cells using Western blot analysis. **(c)** The *in vitro* ubiquitination assay by incubating HectH9, Ubc5a, ubiquitin, and Flag-HAUSP showed that HectH9 mediated K63-lined polyubiquitination of HAUSP WT, but not the $HAUSP^{K443R}$ mutant. **(d)** Western blot analysis showed the decreased K63-linked polyubiquitination levels of HAUSP in H1299 cells under HectH9 knockdown when extracts were immunoprecipitated by anti-HAUSP antibodies followed by blotting with anti-Ub or anti-K63-Ub antibodies. scr-si: scrambled siRNA control. Knockdown of HectH9 significantly decreased the induction of HIF-1α by hypoxia in H1299 cells using Western blot analysis. scr-si: scrambled siRNA control. N: normoxia, H: hypoxia. **(e)-(i)** Knockdown of HectH9 reversed EMT, decreased the expression of HIF-1α targets, and decreased the *in vitro* migration/invasion and *in vivo* metastatic activity in SAS cells. **(j)-(l)** Knockdown of HectH9 reversed EMT, decreased the expression of HIF-1α targets, and decreased the *in vitro* migration/invasion activity in MDA-MB-231 cells. For **(a), (g)**, & **(l)**, data from three independent experiments are expressed as mean ± s.d. For (i), data are presented as the mean ± s.d. (n=6 mice/group). The asterisk (*) indicated statistical significance (P<0.05) between experimental and control treatment, *t*-test. Anti-IgG antibodies were used as negative controls in co-immunoprecipitation assays.

Supplementary Table 1. Univariate analysis for probability of overall survival in 190 patients of resected stage I lung adenocarcinoma

	N (%) of cases	Median (months)	HR	95% CI	*P* value
Age at operation, years					
< 65	98 (51.6)	87.4	1		
> 65	92 (48.4)	76.0	1.071	0.687-1.670	0.762
Gender					
Male	102 (53.7)	87.3	1		
Female	88 (46.3)	72.3	0.739	0.468-1.168	0.193
Extent of pulmonary resection					
Sublobar resection	42 (22.1)	68.3	1		
Anatomical resection	148 (77.9)	–*	0.704	0.426-1.163	0.168
Tumor size					
≤3 cm	113 (59.5)	–*	1		
> 3 to ≤5 cm	77 (40.5)	55.9	1.846	1.183-2.881	0.006
T status					
T1	54 (28.4)	–*	1		
T2a	136 (71.6)	72.3	1.615	0.943-2.766	0.078
HIF-1α overexpression					
No	91 (47.9)	–*	1		
Yes	99 (52.1)	67.5	1.533	0.975-2.410	0.062
HectH9 overexpression					
No	79 (41.6)	–*	1		
Yes	111 (58.4)	57.8	2.930	1.742-4.929	<0.001
HIF-1α and HectH9 co-overexpression					
No	112 (58.9)	–*	1		
Yes	78 (41.1)	63.4	1.927	1.234-3.010	0.003

HR, hazard ratio; CI, confidence interval. *Median survival was not reached.

Supplementary Table 2. Association between HIF-1α and HectH9 expression in lung cancer patients

Variables	HIF-1α overexpression		*P* value
	No	Yes	
HectH9 overexpression			
No	58 (63.7%)	21 (21.2%)	<0.001
Yes	33 (36.3%)	78 (78.8%)	

Supplementary Table 3. Multivariate analysis for probability of overall survival in 190 patients of resected stage I lung adenocarcinoma

	HR	95% CI	*P* value
Tumor size			
≤ 3 cm	1		
> 3 to ≤ 5 cm	1.967	1.144-3.381	0.014
T status			
T1	1		
T2a	1.050	0.548-2.013	0.882
HIF-1α and HectH9 co-overexpression			
No	1		
Yes	2.088	1.329-3.280	0.001

HR, hazard ratio; CI, confidence interval

Supplementary Table 4. Sequence of the oligonucleotides and restriction enzymes used for plasmid construction

Constructions	Sequence (5'→3')	Restriction Enzyme site
HA-HIF-1a 1-400	F: ATAAGAATGCGGCCGCTCACAGCAAAGTTAAAGCATCAGGT	*Not*I
	R: CGCGGATCCAAATGGAGGGCGCCGGCGGC	*BamH*I
HA-HIF-1a 401-826	F: ATAAGAATGCGGCCGCTCACTGGAATACTGTAACTGTGCTT	*Not*I
	R: CGCGGATCCAACAGACTCAAATACAAGAACCTA	*BamH*I
HA-HIF-1a 401-603	F: ATAAGAATGCGGCCGCTCACTGGAATACTGTAACTGTGCTT	*Not*I
	R: CGCGGATCCAAGCCCCAGCCGCTGGAGAC	*BamH*I
HA-HIF-1a 604-826	F: ATAAGAATGCGGCCGCTCAGTTAACTTGATCCAAAGCTCTG	*Not*I
	R: CGCGGATCCAACAGACTCAAATACAAGAACCTA	*BamH*I
Flag-HAUSP WT	F: CCCAAGCTTAACCACCAGCAGCAGCAGCAG	*Hind*III
	R: CGCGGATCCTCAGTTATGGATTTTAATGGCCTT	*BamH*I
Flag-HAUSP 1-450	F: CCCAAGCTTAACCACCAGCAGCAGCAGCAG	*Hind*III
	R: CGCGGATCCTCATGGTGGCAATGTTAGGAATTT	*BamH*I
Flag-HAUSP 1-210	F: CCCAAGCTTAACCACCAGCAGCAGCAGCAG	*Hind*III
	R: CGCGGATCCTCACTTCTTYGAATCCCACGCAACTC	*BamH*I
Flag-HAUSP 1-300	F: CCCAAGCTTAACCACCAGCAGCAGCAGCAG	*Hind*III
	R: CGCGGATCCTCAACAAAGCTCCTGAACATCATG	*BamH*I
Flag-HAUSP 1-400	F: CCCAAGCTTAACCACCAGCAGCAGCAGCAG	*Hind*III
	R: CGCGGATCCTCATGGTGGCAATGTTAGGAATTT	*BamH*I
Flag-HAUSP 1-500	F: CCCAAGCTTAACCACCAGCAGCAGCAGCAG	*Hind*III
	R: CGCGGATCCTCAATAATTGTGCTCAATTGCTTCCTC	*BamH*I
Flag-HAUSP 500-1100	F: CCCAAGCTTGGGGGTCACGATGACGACCTGTCT	*Hind*III
	R: CGCGGATCCTCAGTTATGGATTTTAATGGCCTTTTC	*BamH*I
GST-HAUSP 1-209	F: CGCGGATCCAACCACCAGCAGCAGCAGCAG	*BamH*I
	R:ATAAGAATGCGGCCGCTCACTTCTTTGAATCCCACGCAACTC	*Not*I
GST-HAUSP 210-500	F: CGCGGTCCCACACAGGCTACGTCGGCTTA	*BamH*I
	R:ATAAGAATGCGGCCGCTCAATAATTGTGCTCAATTGCTTCCTC	*Not*I
His-HIF-1a 1-400	F: CGGGGTACCATGGAGGGCGCCGGCGG	*Kpn*I
	R: CGCGGATCCTCACAGCAAAGTTAAAGCATCAG	*BamH*I
His-HIF-1a 401-603	F: CGCGGATCCGCCCCAGCCGCTGGAGA	*BamH*I
	R: CCGCTCGAGTCACTGGAATACTGTAACTGTGC	*Xho*I
HA-HIF-1a 1-80	F: CGGGGTACCATGGAGGGCGCCGGCGG	*Kpn*I
	R: AAGGAAAAAAGCGGCCGCTCAAATATCCAAATCACCAGCATCC	*Not*I
HA-HIF-1a 81-400	F: CGGGGTACCGAAGATGACATGAAAGCACA	*Kpn*I

	R: AAGGAAAAAAGCGGCCGCTCACAGCAAAGTTAAAGCATCAG	*Not*I
HA-HIF-1a 81-180	F: CGGGGTACCGAAGATGACATGAAAGCACA	*Kpn*I
	R: AAGGAAAAAAGCGGCCGCTCATCTTCCTCGGCTAGTTAG	*Not*I
HA-HIF-1a 175-305	F: CGGGGTACCCTAACTAGCCGAGGAAGA	*Kpn*I
	R: AAGGAAAAAAGCGGCCGCTCAGTACTGTCCTGTGGTGAC	*Not*I
HA-HIF-1a 300-400	F: CGGGGTACCGTCACCACAGGACAGTAC	*Kpn*I
	R:AAGGAAAAAAGCGGCCGCTCACAGCAAAGTTAAAGCATCAG	*Not*I
Flag-USP21	F: CCGGAATTCAATGCCCCAGGCCTCTGAGCACCGCCTG	*EcoR*I
	R: CGGGGTACCTCACAGGCACCGGGGTGGCTCCTGCATC	*Kpn*I
HA-CBP 8	F: ATAAGATCTCTGACCGCTTTGTTTATACCTGCAATGAGTGC	*Bgl*II
	R: AAGCGGCCGCCTACCCGGGTGGTGCTGAGGTAGGAGAAGGCAA	*Not*I
HA-CBP 8ΔZnF	F: ATAAGATCTCTTGGGGGCTAGGCCTAGATGATGAGGGCAGC	*Bgl*II
	R: AAGCGGCCGCCTACCCGGGTGGTGCTGAGGTAGGAGAAGGCAA	*Not*I

Supplementary Table 5. Schemes of plasmid constructions for constructs used.

Plasmid	Template	Vector	Restriction enzyme site (F/R)
pCMV-HA-HIF-1a WT	pcDNA3-HA- HIF-1a	pCDNA3	*Not*I / *BamH*I
pCMV-HA-HIF-1a 1-400	pCMV-HA- HIF-1a WT	pCMV-HA	*Not*I / *BamH*I
pCMV-HA-HIF-1a 401-826	pCMV-HA- HIF-1a WT	pCMV-HA	*Not*I / *BamH*I
pCMV-HA-HIF-1a 401-603	pCMV-HA- HIF-1a WT	pCMV-HA	*Not*I / *BamH*I
pCMV-HA-HIF-1a 604-826	pCMV-HA- HIF-1a WT	pCMV-HA	*Not*I / *BamH*I
pCMV-HA-HIF-1a 1-80	pCMV-HA- HIF-1a WT	pCMV-HA	*Kpn*I / *Not*I
pCMV-HA-HIF-1a 81-400	pCMV-HA- HIF-1a WT	pCMV-HA	*Kpn*I / *Not*I
pCMV-HA-HIF-1a 81-180	pCMV-HA- HIF-1a WT	pCMV-HA	*Kpn*I / *Not*I
pCMV-HA-HIF-1a 175-305	pCMV-HA- HIF-1a WT	pCMV-HA	*Kpn*I / *Not*I
pCMV-HA-HIF-1a 300-400	pCMV-HA- HIF-1a WT	pCMV-HA	*Kpn*I / *Not*I
pCMV-HA-HIF-1a S239A	pCMV-HA- HIF-1a WT	pCMV-HA	*Not*I / *BamH*I
pCMV-HA-HIF-1a S282A	pCMV-HA- HIF-1a WT	pCMV-HA	*Not*I / *BamH*I
pCMV-HA-HIF-1a S366A	pCMV-HA- HIF-1a WT	pCMV-HA	*Not*I / *BamH*I
pFlag-CMV2-HAUSP WT	pCMV-HA- HIF-1a WT	pIRES	*Hind*III / *BamH*I
pFlag-CMV2-HAUSP 1-210	pCMV-Flag-HAUSP WT	pFlag-CMV2	*Hind*III / *BamH*I
pFlag-CMV2-HAUSP 1-300	pCMV-Flag-HAUSP WT	pFlag-CMV2	*Hind*III / *BamH*I
pFlag-CMV2-HAUSP 1-400	pCMV-Flag-HAUSP WT	pFlag-CMV2	*Hind*III / *BamH*I
pFlag-CMV2-HAUSP 1-450	pCMV-Flag-HAUSP WT	pFlag-CMV2	*Hind*III / *BamH*I
pFlag-CMV2-HAUSP 1-500	pCMV-Flag-HAUSP WT	pFlag-CMV2	*Hind*III / *BamH*I
pFlag-CMV2-HAUSP 500-1100	pCMV-Flag-HAUSP WT	pFlag-CMV2	*Hind*III / *BamH*I
pFlag-CMV2-HAUSP 1-450 K420R	pFlag-CMV2-HAUSP 1-450	pFlag-CMV2	*Hind*III / *BamH*I
pFlag-CMV2-HAUSP 1-450 K439R	pFlag-CMV2-HAUSP 1-450	pFlag-CMV2	*Hind*III / *BamH*I
pFlag-CMV2-HAUSP 1-450 K443R	pFlag-CMV2-HAUSP 1-450	pFlag-CMV2	*Hind*III / *BamH*I
pFlag-CMV2-HAUSP K443R	pFlag-CMV2-HAUSP WT	pFlag-CMV2	*Hind*III / *BamH*I
pFlag-CMV2-Usp21	gDNA	pFlag-CMV2	*EcoR*I / *Kpn*I
pGEX4T1-HAUSP 1-209	pCMV-HA- HIF-1a WT	pGEX4T1	*BamH*I / *Not*I
pGEX4T1-HAUSP 210-500	pCMV-HA- HIF-1a WT	pGEX4T1	*BamH*I / *Not*I
pET32a-His-HIF-1a 1-400	pcDNA-HA- HIF-1a WT	pET32a	*Kpn*I / *BamH*I
pET32a-His-HIF-1a 401-603	pcDNA-HA- HIF-1a WT	pET32a	*BamH*I / *Xho*I
pCMV-HA-CBP CH3	pFlag-CMV2-CBP 1680-1892	pFlag-CMV2	*Bgl*II / *Not*I
pCMV-HA-CBP CH3ΔZZ	pFlag-CMV2-CBP 1680-1892	pFlag-CMV2	*Bgl*II / *Not*I
pCMV-HA-CBP CH3ΔZZ-TAB2	pFlag-CMV2-CBP 1680-1892	pFlag-CMV2	*Bgl*II / *Not*I
pCMV-HA-CBP 8 C1710S	pCMV-HA-CBP 1702-1892	pFlag-CMV2	*Bgl*II / *Not*I

pCMV-Flag-CBP	pIRES-Flag-CBP	pFlag-CMV2	*Hind*III / *BamH*I
pCMV-Flag-CBPΔZZ	pIRES-Flag-CBP	pFlag-CMV2	*Hind*III / *BamH*I
pCMV-Flag-CBPΔZZ-TAB2	pIRES-Flag-CBP	pFlag-CMV2	*Hind*III / *BamH*I
pFlag-CMV6-Usp12	gDNA	pFlag-CMV6	*SgfI/Mlu*I
pFlag-CMV6-Usp14	gDNA	pFlag-CMV6	*SgfI/Mlu*I
pFlag-CMV2-Usp21	gDNA	pFlag-CMV2	*EcoR*I / *Kpn*I

Supplementary Table 6. List of proteins tested by and characteristics of the corresponding antibodies

Protein	Assay	Origin	Dilution	Incubation period
HIF-1α	WB	#610958, BD Biosciences	1:250	4℃, Overnight
HIF-1α	IP	#610958, BD Biosciences	5 μl	4℃, Overnight
HIF-1α	CHIP	PA3-16521, Thermo scientific	5 μl	4℃, Overnight
E-cadherin	WB	#4065, Cell signaling	1:500	4℃, Overnight
vimentin	WB	V-6630, Sigma-Aldrich Corp.	1:1000	4℃, Overnight
N-cadherin	WB	#610921, BD Biosciences	1:500	4℃, Overnight
plakoglobin	WB	Ab11506, Abcam	1:1000	4℃, Overnight
GLUT-1	WB	#07-1401, Millipore	1:1000	4℃, Overnight
VEGF	WB	19003-1-AP, Proteintech	1:1000	4℃, Overnight
β-actin	WB	A-5441, Sigma-Aldrich Corp.	1:10000	4℃, Overnight
Flag	WB	F3165, Sigma-Aldrich Corp.	1:1000	4℃, 1 hour
Flag	IP	F3165, Sigma-Aldrich Corp.	5 μl	4℃, Overnight
HA	WB	#2367, Cell signaling Technology, Inc.	1:1000	4℃, 1 hour
HA	IP	#2367, Cell signaling Technology, Inc.	5 μl	4℃, Overnight
HA	IP	05-904, Millipore	5 μl	4℃, Overnight
TWIST	WB	Sc-15303, Santa cruz Biotechnology, Inc.	1:500	4℃, Overnight
HAUSP	WB	A300-033A, Bethyl	1:1000	4℃, Overnight
HAUSP	IP	A300-033A, Bethyl	5 μl	4℃, Overnight
USP21	WB	BML-PW0585, Enzo	1:1000	4℃, Overnight
USP21	IP	Sc-79305, Santa cruz Biotechnology, Inc.	5 μl	4℃, Overnight
Ubiquitin	WB	BML-PW8810, Enzo	1:1000	4℃, Overnight
Ubiquitin, Lys48-Specific	WB	#05-1307, Millipore	1:1000	4℃, Overnight
Ubiquitin, Lys63-Specific	WB	#05-1308, Millipore	1:500	4℃, Overnight
Ubiquitin, Lys63-Specific	WB	#05-1308, Millipore	1:500	4℃, Overnight
VHL	WB	#2738, Cell signaling Technology, Inc.	1:1000	4℃, Overnight
HectH9	WB	#5695, Cell signaling Technology, Inc.	1:1000	4℃, Overnight
TRAF6	WB	TA300591, OriGene Technologies, Inc.	1:1000	4℃, Overnight
HectH9	IHC	NBP1-83127, Novus	1:500	4℃, Overnight
HIF-1	IHC	Sc-10790, Santa Cruz	1:250	4℃, Overnight
CBP	WB	GTX307050, Genetex	1:20	4℃, Overnight
CBP	CHIP	#7389, Cell signaling Technology, Inc.	5 μl	4℃, Overnight
CDK8	WB, CHIP	Sc-1521, Santa cruz Biotechnology, Inc.	10 μl	4℃, Overnight

AFF4	CHIP	A302-538A, Bethyl	5 μl	4℃, Overnight
H3K56Ac	CHIP	#17-10259, Millipore	3 μl	4℃, Overnight
H2AZ	CHIP	PA5-21923, Thermo scientific	3 μl	4℃, Overnight
H4K5Ac	CHIP	#17-10045, Millipore	3 μl	4℃, Overnight
H3K4Ac	WB	#07-539, upstate	1:250	4℃, Overnight
H3K9Ac	WB	#06-942, upstate	1:500	4℃, Overnight
H3K14Ac	WB	#06-911, millipore	1:500	4℃, Overnight
H3K56Ac	WB	#17-10259, Millipore	1:500	4℃, Overnight
H4K5Ac	WB	ab51997, abcam	1:500	4℃, Overnight
H4K12Ac	WB	ab61238, abcam	1:500	4℃, Overnight
H3	WB	#9715, Cell signaling Technology, Inc.	1:500	4℃, Overnight
H2AZ	WB	#2718, Cell signaling Technology, Inc.	1:500	4℃, Overnight
H2A	WB	#2578, Cell signaling Technology, Inc.	1:500	4℃, Overnight
H3K18Ac	WB	ab1191, abcam	1:500	4℃, Overnight
H3K27Ac	WB	ab4729, abcam	1:500	4℃, Overnight
H2AK5Ac	WB	ab1764, abcam	1:500	4℃, Overnight
MED1	WB	A300-793A, Bethyl	1:500	4℃, Overnight
RNA pol II	WB	A300-653A, Bethyl	1:500	4℃, Overnight
MED26	WB	Sc-48776, Santa cruz Biotechnology, Inc.	1:500	4℃, Overnight
CDK8	WB	Sc-1521, Santa cruz Biotechnology, Inc.	1:500	4℃, Overnight
AFF4	WB	A302-539A, Bethyl	1:500	4℃, Overnight
CBP	IP	GTX307050, Genetex	50 μl	4℃, Overnight
YY1	CHIP	ab12132, abcam	5 μl	4℃, Overnight
HectH9	IP	R960-25, ThermoFisher scientific	5 μl	4℃, Overnight

Abbreviations: WB, Western blot; IP, Immunoprecipitation; mmab, mouse monoclonal antibody; rmab, rabbit monoclonal antibody; rpab, rabbit polyclonal antibody; gpab, goat polyclonal antibody

Supplementary Table 7. Sequence of the oligonucleotides for real-time PCR

Target	Sequence (5'→ 3')
HIF-1a	F: TTTTTCAAGCAGTAGGAATTGGA
	R: GTGATGTAGTAGCTGCATGATCG
Twist1	F: AGCTACGCCTTCTCGGTCT
	R: CCTTCTCTGGAAACAATGACATC
HectH9	F: TTGGACCGCTTCGATGGAATA
	R: TGAAGTTCAACACAGCCAAGAG
TRAF6	F: GATGCAGAGGAATCACTTGGC
	R: GGTCTTGTCTTACAAGGCGAC
RNF8	F: CCCGGCTTCTTCGTCACAG
	R: ACCTCGCACCCATCTTCCA
Siah2	F: CATCAGGAACCTGGCTATGG
	R: GGACGGTATTCACATATGTC
c.IAP1	F: AGAGGAGAAGGAAAAACAAGCT
	R: ATCCAGGATAGGAAGCACACA
c.IAP2	F: TTCCACACACTCATTACTTCC
	R: TCTGGAGTTTACAGGATTTGATG
VEGF	F: GTACCCTGATGAGATCGAGT
	R: GTGATGTTGGACTCCTCAGT
mTwist1	F: AGCGGGTCATGGCTAACG
	R: AGCTCGTCGCTCTGCAGG
mGlut1	F: CGGAACTCCATGCTGATGATG
	R: CCACATACATGGGCACAAAGC
Vegf	F: AAGCCAGCACATAGGAGAGATGA
	R: CTGTCTTTCTTTGGTCTGCATTCA
m18S	F: TTCGTATTGCGCCGCTAGA
	R: CTTTCGCTCTGGTCCGTCTT
HAUSP K444R	F: CCCAATTGGTGAAAGAGCTTA
Typing primer	R: CTGGAAACTCAAACCTGTTCAA

Supplementary Table 8. Sequence of the oligonucleotides for ChIP assays

Target	Sequence (5’→ 3’)
Twist1 (-1136~-1024)	F: CCTCTTTGGGGCTCTTCGTT
	R: TCTAGGGCATCCAGTGGACA
Twist1 (-891~-767)	F: TGAAATGGCCACAGGGTCTC
	R: TTCGGTGGAAGGAAACCCAG
Twist1 (-509~-399)	F: CACTTTTCTTGGCATGCCCC
	R: GGTGATGTCTCATCTCGCCC
Twist1 (-98~-29)	F: GGGACTGGAAAGCGGAAACT
	R: TGTCATTGGCCTGACGTGAG
Twist1 (647~800)	F: GTCCGCAGTCTTACGAGGAG
	R: TTGAGGGTCTGAATCTTGCTC
Twist1 (1483~1677)	F: AACAGCCGCAGAGACCTAAA
	R: CACGCCCTGTTTCTTTGAAT
Twist1 (3536~3640)	F: GGAAGGCGATTATGTGTTGG
	R: ATGCTTTCCCTCATCCTCCT
Twist1 (4770~4897)	F: CATGGTTTGGCAAAGTCAGA
	R: ATCATGAGAGCGGGACAAAC
VEGF (-992~-900)	F: GCCAGACTCCACAGTGCATA
	R: CTGAGAACGGGAAGCTGTGT
VEGF (-889~-787)	F: TTGGTGCCAAATTCTTCTCC
	R: GCCTGCAGACATCAAAGTGA
VEGF (-526~-470)	F: GCGTCTTCGAGAGTGAGGAC
	R: CACACGCACACACTCACTCA
VEGF (-340~-285)	F: AAAGAGGGAACGGCTCTCAG
	R: AGGGAGCAGGAAAGTGAGGT
VEGF (407~517)	F: ACAGGGGCAAAGTGAGTGAC
	R: CTGTCTGTCTGTCCGTCAGC
VEGF (1578~1649)	F: GCTTGCTGTCACTGCCACT
	R: AGCAATCCACCCCAAAACTT
VEGF (3063~3146)	F: CTAGCAGGGTCTGGTGTTCC
	R: CTCCAGCTCTCACCAACTCC
VEGF (4803~4935)	F: TGGATCCTCCCATTTCTCTG
	R: CCACGCCAGATTTAGGTCAG
Glut1 (-3247~-3093)	F: CATTTGGGAGCCTCTGTGAT
	R: GAGAAGGCCCCAGGAAATAG

Glut1 (-2154~-1980)	F: TGGGAAAAGGCATAGACTGG
	R: ATGCACGAATGAGTGAGCAG
Glut1 (-1062~-925)	F: CTTGAGCCCAGGAGTTTGAG
	R: GGAGAGGTGCAATTTCCAGA
Glut1 (-774~-634)	F: TGGTGAAACCCCGTCTCTAC
	R: CACGATCTCGGCTCACTGTA
Glut1 (-501~-370)	F: TGGTTCAAACCCGAGGTCTA
	R: GGGACGCCTTCCTCTACTTC
Glut1 (-188~-46)	F: TCCATGCAGTAGACGCTGTT
	R: TGGAAAGCTGGACTGGAGTT
Glut1 (304~426)	F: CTGGCAAGAGGCAAGAGGTA
	R: ACTCCCACTGCGACTCTGAC
Glut1 (1464~1570)	F: CCTGCAGGGCATCTTTGTAG
	R: GGAGGAAAGGAGGTGGAAAG
Glut1 (3018~3102)	F: TTTCCCCTCCAAATCTTGTG
	R: TGCAGGTCAAATCAGCAGTC
Glut1 (5164~5294)	F: GGAGCCTTGGTTTCCTTCTC
	R: CCCAGACTGGCCTTACAAAG
E-cadherin (-1137~-992)	F: AAACTGAGGCTTTGGGAGGT
	R: TTCTGAACTCAGGCGATCCT
E-cadherin (-767~-640)	F: CGCCTGTAGTCCCAGCTACT
	R: GACGGAGTCTCGCTCTGTCT
E-cadherin (-582~-479)	F: ATGGCTCACACCTGAAATCC
	R: GCGCTGTGTCTCCCTGTATT
E-cadherin (-287~-212)	F: GAGTGAGACCCCATCTCCAA
	R: GGGCTTTTACACTTGGCTGA
N-cadherin (-1145~-1059)	F: TGCACTCTCAAACTCCCAGA
	R: CTCCCACCCTTGAGGATGTA
N-cadherin (-871~-765)	F: TTAGGCTCCCAAGGAGACAC
	R: GGGCAGGAACTTGATTGGTA
N-cadherin (-616~-499)	F: CTACAGCCGCAGCTTGGT
	R: CCGCTCCCCTCTCCTATTC
N-cadherin (-242~-132)	F: CCGGAGAACAGTCTCCAACT
	R: ACCCACAAAGAGCAGCAGTC
KDM3A (-1257~-1032)	F: GCCCAGTATTTTCAAGCTTCC
	R: CCAGCTGAAAGAAGGCAGAA
KDM3A (-495~-333)	F: CCAGTAACTCCACGCCTTTC

	R: GGAGGAGCGATAGTGCTCTG
KDM3A (-71~155)	F: TGTGAGGCAACAAATGGAAA
	R: TCATTGGCTGAAACACAGGA
Twist1 (-3619~-3454)	F: CCCTCTGGAGTGTTCAAAGC
	R: CTGTTTGTTGGCTCCGAGTT
Twist1 (-1289~-1125)	F: TAAGGGATGGACCTGAAACG
	R: GCCCCAAAGAGGGTGTTAAT
Twist1 (-707~-534)	F: CGTCAGACTGGGTCGTTGTA
	R: TCAGGTAGACGAACCCCTTG
VEGF (-3181~-2998)	F: TTGGAGGTGACAGGACATCA
	R: CAGTTCAGAAGGAGCCAAGG
VEGF (-2136~-2031)	F: GGCTCTTTTAGGGGCTGAAG
	R: GTAGACATCTTGGGGCAGGA
VEGF (-222~-103)	F: TTCAGGCTGTGAACCTTGG
	R: GAGCCTCAGCCCTTCCAC
Glut1 (-4460~-4346)	F: CAGGGAGGAGGATGAATGAA
	R: CAGTCAATCCCGTGGACTTT
Glut1 (-1999~-1826)	F: CTGCTCACTCATTCGTGCAT
	R: CTGGTTCTCCCAAGTGGAAA
Glut1 (-1594~-1440)	F: GCAGGACTGGAGGACAAAAG
	R: CTATGCTCAGCCTGGAAAGC
β-ACTIN (-1099~-931)	F: TGACAAGGACAGGGTCTTCC
	R: CACCGTCCGTTGTATGTCTG
β -ACTIN (-671~-458)	F: GACTTCTAAGTGGCCGCAAG
	R: CTGCAGAAGGAGCTCTTGGA
β -ACTIN (-394~-173)	F: AGTGCCCAAGAGATGTCCAC
	R: AGAGCGAGAGCGAGATTGAG
β -ACTIN (333~521)	F: CGGGGTCTTTGTCTGAGC
	R: GCAGTTAGCGCCTTGAGTC
β -ACTIN (1326~1534)	F: AGAAAATCTGGCACCACACC
	R: AACGGCAGAAGAGAGAACCA
β -ACTIN (2998~3168	F: AAACTGGAACGGTGAAGGTG
	R: AGAGAAGTGGGGTGGCTTTT
GAPDH (-1192~-1029)	F: GGCTGGTGTCAGGTTATGCT
	R: TCTTCTGGTAGGAGGGCAGA
GAPDH (-772~-604)	F: AGGTTTCCAGGAGTGCCTTT
	R: CACGGAAGGTCACGATGTC

GAPDH (-273~-27)	F: CAATTCCCCATCTCAGTCGT
	R: TAGTAGCCGGGCCCTACTTT
GAPDH (532~695)	F: GGGTCTTTGCAGTCGTATGG
	R: TTTCTGGGGACTAGGGGAAG
GAPDH (1432~1647)	F: CCCACACACATGCACTTACC
	R: CCCACCCCTTCTCTAAGTCC
GAPDH (2815~3190)	F: CTCCCACCTTTCTCATCCAA
	R: TGGACTGTGGTCTGCAAAAG

Supplementary Table 9. Lentivirus used in RNAi experiments

Target	Sequence	Clone ID
Scrambled control	CCTAAGGTTAAGTCGCCCTCG	
HAUSP (USP7)	CGTGGTGTCAAGGTGTACTAA	TRCN0000010845
HectH9 (HUWE1)	CGACGAGAACTAGCACAGAAT	TRCN0000073306
TRAF6	GCCACGGGAAATATGTAATAT	TRCN0000007348

Supplementary Notes

(: containing full blots of regular figures)

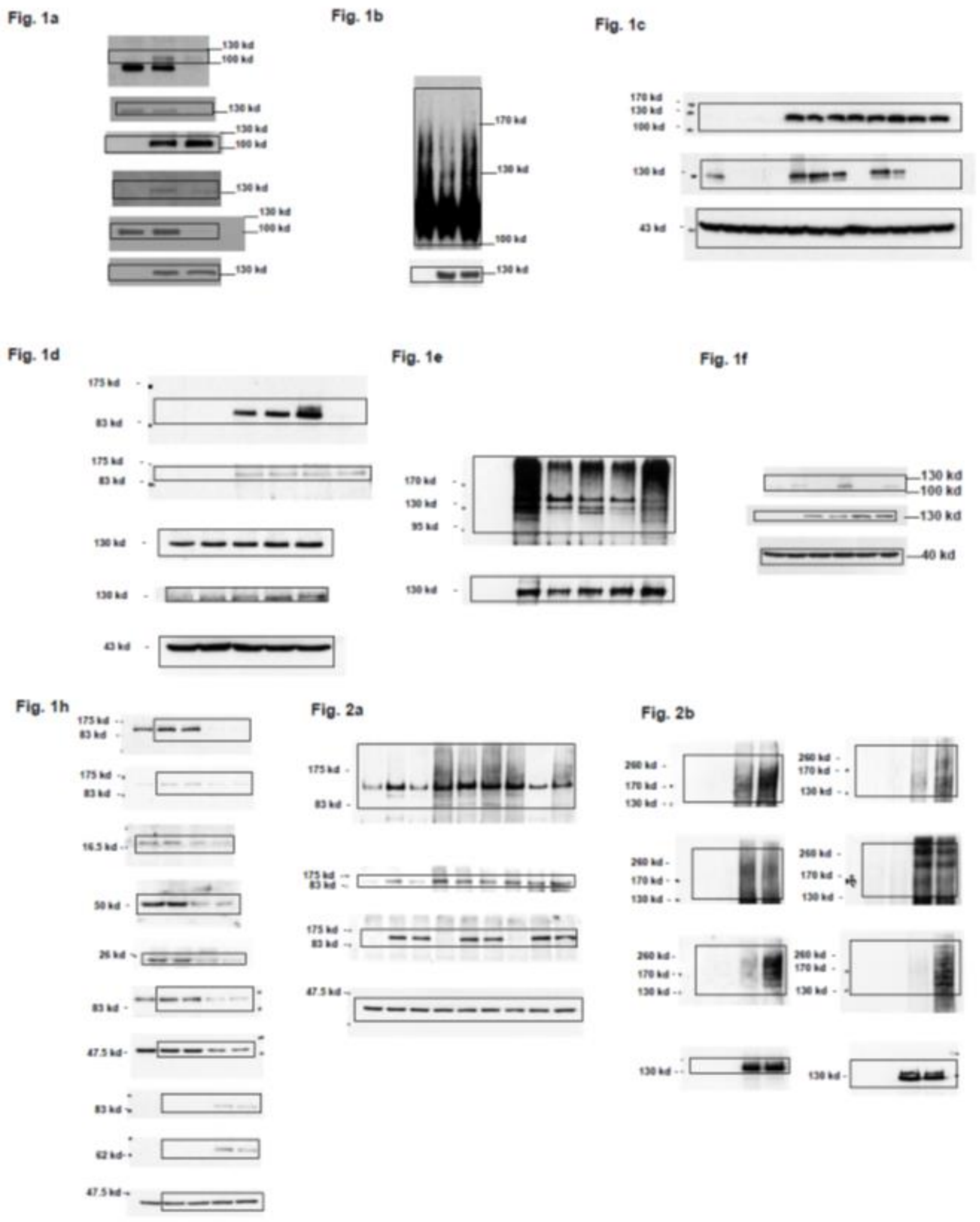

Fig. 2c

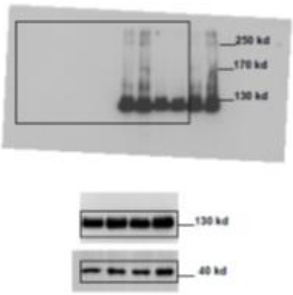

Fig. 2e

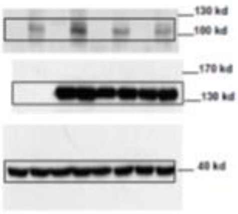

Fig. 2f

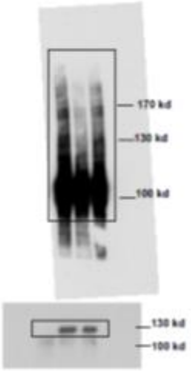

Fig. 3a

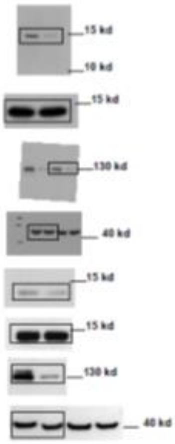

Fig. 3b

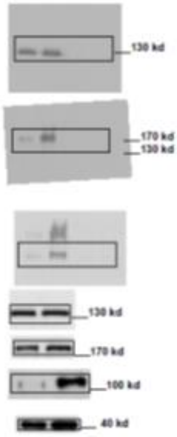

Fig. 3c

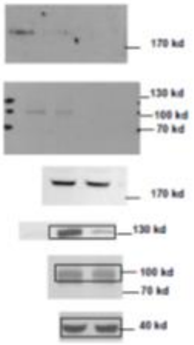

Fig. 3d

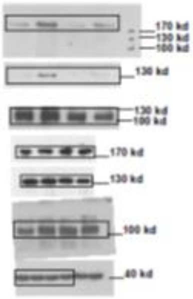

Fig. 3e

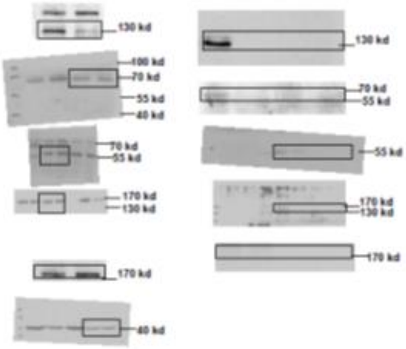

Fig. 3f

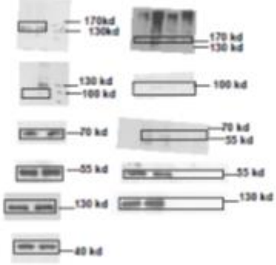

Fig. 3g

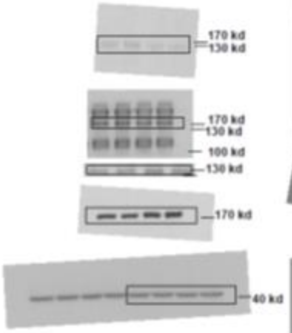

Fig. 5b

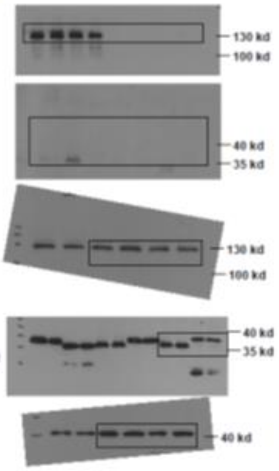

Fig. 5c

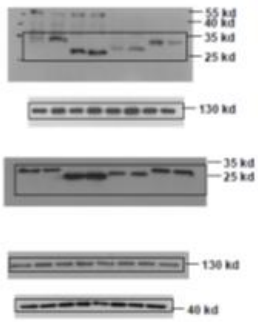

Fig. 5d

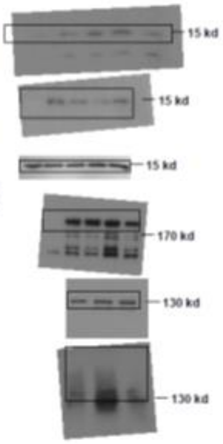

Fig 8a

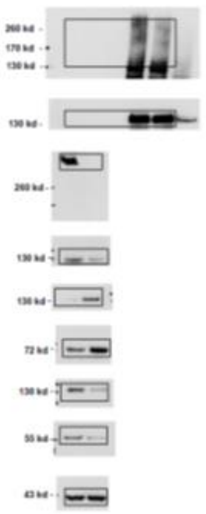

Fig. 5e

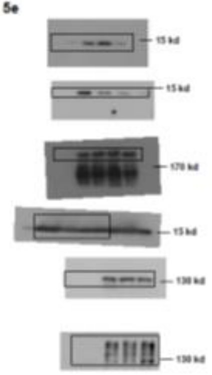

Fig. 7d

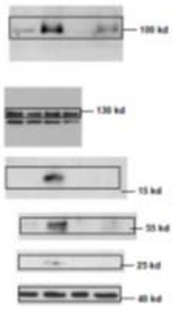

Fig. 7e

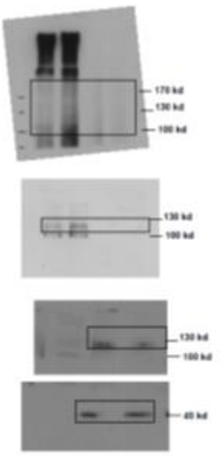

Supplement: Supplementary Information — Supplementary Figures 1-7, Supplementary Tables 1-9, Supplementary Notes. [file ncomms13644-s1.pdf]
